# The PKCι-β-arrestin2 axis disrupts SORLA retrograde trafficking, driving its degradation and amyloid pathology in Alzheimer’s disease

**DOI:** 10.1186/s13024-025-00865-6

**Published:** 2025-06-23

**Authors:** Hasibur Rehman, Shun Yan, Shalini Saggu, Mae Aida, Fang Zhang, Yang Shu, Alexis Jones, Amy Trang, Emily Dew, Wenbo Zhi, Emily T. Claeboe, Anthony J. Baucum, Guangyu Wu, Kai Jiao, Qin Wang

**Affiliations:** 1 Department of Neuroscience and Regenerative Medicine, Medical College of Georgia at Augusta University, 1120 15 Street, Augusta, GA 30912 USA; 2 Center for Biotechnology and Genomic Medicine, Medical College of Georgia at Augusta University, Augusta, GA 30912 USA; 3 Department of Cell, Developmental and Integrative Biology, University of Alabama at Birmingham, Birmingham, AL 35294 USA; 4 Department of Pharmacology and Toxicology, Indiana University School of Medicine, Indianapolis, IN 46202 USA; 5 Stark Neurosciences Research Institute, Indiana University School of Medicine, Indianapolis, IN 46202 USA; 6 Department of Pharmacology and Toxicology, Medical College of Georgia at Augusta University, Augusta, GA 30912 USA; 7 Present Address: Structural Biology Program, Memorial Sloan Kettering Cancer Center, New York, NY 10065 USA

**Keywords:** SORLA, PKCι/λ, βarrestin2, Alzheimer’s disease, Protein–protein interaction, Trafficking, Degradation, Treatment, Aβ production

## Abstract

**Background:**

Variants of *SORL1* have been associated with both late and early onset of Alzheimer’s disease (AD). *SORL1* encodes the sorting-related receptor with A repeat (SORLA) protein, which belongs to the VPS10 receptor family. SORLA protects against AD pathogenesis through its sorting function, and reduced SORLA levels have been consistently observed in sporadic AD. Although the importance of SORLA in AD pathogenesis is well recognized, how it can be targeted for AD treatment remains to be established, owing to the inadequate understanding of its regulation by intracellular signaling.

**Methods:**

We employed combined biochemical, cell biological, and pharmacological approaches to investigate how SORLA trafficking and stability are regulated. Additionally, we used an AD mouse model, postmortem tissue samples, and iPSC-derived neurons to examine the functional outcomes of this regulation.

**Results:**

We identified a novel direct interaction between SORLA and β-arrestin2 (βARR2), which impedes the interaction of SORLA with the retromer complex, thus reducing the retrograde trafficking of SORLA. βARR2 promotes the interaction between SORLA and the ESCRT0 complex, leading to the lysosomal localization and degradation of SORLA. We also found that PKCι/λ induces SORLA phosphorylation and enhances its interaction with βARR2, promoting SORLA degradation. Importantly, blocking PKCι/λ with auranofin disrupts the SORLA-βARR2 interaction, elevates SORLA levels, decreases amyloidogenic processing of APP, and improves cognition in the *App*^*NL−G−F/NL−G−F*^ AD mouse model. Furthermore, PKCι is hyperactive in human AD brains, and auranofin reduces Aβ production in AD iPSC-derived neurons through increasing SORLA levels.

**Conclusion:**

Our study reveals the PKCι/λ-βARR2 axis as a key molecular mechanism that disrupts SORLA retrograde trafficking and drives its degradation. Our findings represent the first evidence that SORLA levels can be pharmacologically manipulated through blocking PKCι/λ to reduce Aβ production and alleviate AD-related phenotypes. Notably, repurposing auranofin, an FDA-approved drug for rheumatoid arthritis, may offer the potential for AD treatment.

**Supplementary Information:**

The online version contains supplementary material available at 10.1186/s13024-025-00865-6.

## Background

Alzheimer’s disease (AD) has a strong genetic basis with multiple genetic risk factors identified. A better understanding of how these factors are regulated in the brain could provide key information for identification of novel targets for personalized preventive/therapeutic strategies. However, this knowledge remains limited. One of the top genetic risk factors for AD is *SORL1*, which encodes the sorting-related receptor with A-repeat (SORLA) protein, a member of the vacuolar protein sorting 10 (VPS10) receptor family that traffics between various endocytic compartments [1]. Genome-wide association studies (GWAS) have repeatedly identified associations between *SORL1* variants and late-onset AD [2–4]. Moreover, nonsense and missense mutations in *SORL1* cause autosomal dominant early-onset AD [5–7], supporting an etiological role of this gene in AD. Genetic studies using mouse models also underscore the pivotal role of SORLA in AD pathogenesis [8–10]. Despite its importance in AD, our knowledge of how SORLA is regulated by intracellular signaling molecules and the relevance of this regulation to AD is surprisingly scarce.

Newly synthesized SORLA is delivered to the plasma membrane through the secretory pathway. Cell surface SORLA undergoes clathrin-dependent endocytosis to translocate to early endosomes [11], and then shuttles cargo between endosomes and the trans Golgi network (TGN). Retrograde trafficking of SORLA requires the multimeric adaptor complex retromer that binds to the FANSHY motif of the SORLA C-terminal tail [12, 13], whereas the anterograde trafficking of SORLA involves GGA1/2 [14, 15]. SORLA can also move cargo from endosomes to the cell surface [16] or lysosomes [9]. Through its sorting function, SORLA protects against AD pathogenesis in multiple ways. SORLA directly interacts with both amyloid precursor protein (APP) and retromer to guide APP sorting to the TGN, thus reducing the amyloidogenic processing of APP by BACE1 in endosomes [8, 11, 13, 17, 18]. SORLA also interacts with SNX27 to promote APP recycling to the cell surface, enhancing non-amyloidogenic processing [16]. Moreover, SORLA can facilitate Aβ clearance by promoting its lysosomal degradation [9]. In addition to its role in regulating Aβ metabolism, SORLA can modulate neuronal endosome morphology [19]. Endosome enlargement and dysfunction occur early in AD [20–23]. Loss of *SORL1* causes neuronal endosome enlargement independent of amyloidogenic APP processing in human induced pluripotent stem cells (iPSCs) [24]. Therefore, SORLA serves as a critical guard not only to suppress Aβ levels but also to maintain normal endosome morphology and function. Consistently, reduced SORLA expressions or functions have been linked to sporadic AD [3, 4, 25]. Whether increasing SORLA levels could serve as a therapeutic strategy to mitigate the progression of AD-related deficits remains unexplored.

In the current study, we identified a novel direct interaction between SORLA and βarrestin2 (βARR2), a ubiquitously expressed protein that belongs to the arrestin family. Arrestins were originally identified as key regulators of G protein-coupled receptors (GPCRs) and have then been established as multi-functional adaptors that modulate endocytic trafficking and intracellular signaling of many membrane proteins/receptors [26–28]. βARR2 levels are elevated in AD [29] and a genetic variation of *ARRB2* (the gene encoding βARR2) is associated with late-onset AD in a human population [30], suggesting *ARRB2* as a potential genetic risk factor for AD. βARR2 can modulate both amyloid [29] and tau [31] metabolism. The primary objective of this study was to determine the molecular mechanism and pathophysiological significance of βARR2-mediated regulation of SORLA in AD. The results we obtained shed new light on how two genetic risk factors interact to impact AD pathophysiology. Furthermore, our study suggests that elevating SORLA levels, as achieved in our case by blocking PKCι/λ to disrupt the SORLA-βARR2 interaction, is an effective strategy to reduce Aβ production and alleviate AD-related phenotypes.

## Methods

### Antibodies, chemicals, and reagents

All reagents and drugs were sourced from either Sigma-Aldrich or Fisher Scientific, unless otherwise specified. A complete list of commercial antibodies, including vendors, catalog numbers, and usage, is provided in Supplementary Table S1. Small interfering RNAs (siRNAs) against *SORL1* or *Hrs* (TriFecta Kit DsiRNA Duplex) were purchased from Integrated DNA Technologies (IDT, Iowa).

### DNA constructs

The Myc-tagged human SORLA construct (pCMV6-Entry-SORL1) was purchased from Origene (RC214154), with the Myc tag inserted at the C-terminal end of SORLA. To generate the SNAP-tagged SORLA construct, the human *SORL1* cDNA was PCR amplified using high fidelity Taq polymerase and subcloned into the pSNAPf vector (New England Biolabs) at the AgeI and EcoRI restriction sites, maintaining an in-frame fusion with the C-terminal SNAP tag. The mutant Myc-SORLA construct, in which Ser at positions 2178 and 2179 were mutated to Ala (S2178A/S2179A), was generated by synthesizing and PCR-amplifying a *SORL1* cDNA fragment containing the desired mutations. This mutated fragment was then used to replace the corresponding wild-type region in the pCMV6-Entry-SORL1 construct via subcloning at the ApaI and MluI restriction sites. To generate the SORLA-C construct, a *SORL1* cDNA fragment encoding the signal peptide (a.a 1–34) and the C terminal sequence (a.a. 2111–2214) was synthesized, PCR-amplified, and subcloned into the pCDNA3.1 vector at the BamHI and XbaI restriction sites. The GST-fused VPS26 construct was generated by PCR amplification of VPS26 cDNA, followed by subcloning into the pGEX-2 T vector at the BamHI and EcoRI restriction sites. All newly generated constructs were verified by sequencing. GST-fused βARR2 [32] and SMAD [33], and GFP-tagged βARR2 [32], and Flag-tagged WT and mutant VPS26 constructs [13] were described previously. Constitutively active PKCι/λ-A120E [34] and PKCι/λ-CAT [35] constructs were obtained from Addgene.

### Animals

The *Sorl1*^*−/−*^ mouse line, a generous gift from Dr. Thomas Willnow (Max Delbruck Center), was generated as described in [8]. The *App*^*NL−G−F/NL−G−F*^ knock-in line (referred to as AppKI), generously provided by Dr. Takaomi Saido (Riken Center for Brain Science), carries the APP Swedish (KM670/671NL), Iberian (I716F), and Arctic (E693G) mutations and was generated as described in [36]. The generation of *Arrb2*^*−/−*^ mice was described previously in [37]. All mice used in this study were maintained on a C57BL/6 background, with genotypes determined shortly after weaning. To ensure randomization in testing, mice from each litter with wild-type (WT), knock-in (KI), or knockout (KO) genotypes were randomly assigned to either the vehicle or various drug-treated groups. Unless otherwise specified, all experiments used littermate mice, including both males and females.

Mice were housed in the Augusta University animal care facility under standard environmental conditions (12-h light–dark cycle, temperature: 22 ± 1 °C, humidity: 50%) in filtered cages with ad libitum access to food and water. All procedures had been approved by the Augusta University Institutional Animal Care and Use Committee (approval #2021–1061) and the UAB Institutional Animal Care and Use Committee (IACUC-22176). The ARRIVE guidelines were adhered to in the preparation of this manuscript [38].

### Mouse treatment

For chronic treatment with auranofin, 15-week-old AppKI mice were randomly assigned to two groups (N = 14–15 per group). One group received auranofin (5 mg/kg) dissolved in 5% DMSO diluted in distilled water, while the other group received only the vehicle (5% DMSO) diluted in water. Treatments were administered daily via oral gavage (200 µl) for 7 weeks. Following the final treatment, a 3-day washout period was implemented to minimize residual drug effects and ensure an accurate assessment of treatment outcomes. After the washout, behavioral testing was performed, followed by euthanasia for histopathological and biochemical analyses.

In vivo inhibition of PKCι/λ in WT and *Arr2b*^*−/−*^ mice (3–5 months old) was achieved through intraperitoneal (i.p.) administration of CRT0066854 or vehicle at a dose of 5 mg/kg, twice daily for two weeks. Twenty-four hours after the final injection, mouse cerebral cortices were isolated and homogenized. SORLA levels in the total lysates were measured by Western blot analysis. To evaluate the impact of PKC inhibitors on the SORLA-βARR2 interaction, 3–5-month-old WT mice were treated with vehicle, CRT0066854 (5 mg/kg, i.p.), or Go6976 (5 mg/kg, i.p.), administered three times at 12-h intervals. Two hours following the final injection, cerebral cortices were dissected, homogenized, and subjected to co-immunoprecipitation (co-IP) analysis.

### Behavioral tests

#### Open field test (OF)

General activity and locomotion were assessed using the open-field test. The apparatus consisted of a square, opaque, Plexiglas enclosure, where mice were placed in the center of the arena and monitored for 4 min, as previously described [39]. The setup was thoroughly cleaned between trials. Data collected included the percentage of time spent in center versus enclosed areas, and total distance traveled recorded using EthoVision 11.5 video tracking software (Noldus, NL).

#### Elevated zero maze test (EZM)

The elevated zero maze (EZM) assesses anxiety-like and risk-taking behavior. It features an elevated"O"shaped platform with two enclosed and two open sections. Each animal undergoes a 4-min trial, starting in an open section, facing away from the closed section, as described earlier [39]. The location of the animal is classified based on whether all four paws are in an open or closed section, and the percentage of time spent in each area is recorded.

#### Passive avoidance test (PA)

The passive avoidance test was conducted using a two-compartment box, as previously described [40], to assess associative learning. Mice were placed in a lighted chamber, and after 30 s, the door to the darkened chamber was opened. Upon entering the darkened chamber, the door closed, and a brief foot shock (0.5 mA for 2 s) was delivered. After spending 30 s in the dark chamber, mice were returned to their home cage. The test was repeated 24 h later without a foot shock, and the latency to enter the darkened chamber was recorded.

### Preparation of total lysates from mouse brains

Mouse cerebral cortices were homogenized in ice-cold 1.5X lysis buffer (75 mM Tris, pH 6.8, 15% glycerol, and 3% SDS) containing a protease inhibitor cocktail from Fisher (#PI78444). Homogenates were incubated at 4 °C for 30 min and centrifuged at 15,000 rpm for 30 min, and the supernatants were collected as loading samples. Protein concentrations were determined using the DC-protein assay (Bio-Rad, #500–0115).

### Quantitative reverse transcription polymerase chain reaction (qRT-PCR) analysis

Total RNA was extracted from WT or *Arr2b*^*−/−*^ cortical tissues using the Qiagen RNeasy kit, and cDNA synthesis was performed with the Thermo Fisher Scientific Reverse Transcription Kit. Quantitative PCR was carried out using SYBR Green Master Mix on a Bio-Rad CFX96 Real-Time PCR system. Primers for housekeeping genes (e.g., GAPDH or β-actin) were used for normalization. Relative expression was calculated using the 2^-ΔΔCt method.

### Human brain sample preparations

Human prefrontal cortex samples (Table S2) were obtained from the University of Washington Alzheimer’s Research Center under Institutional Review Board-approved human study protocols. For total protein extraction, tissues were homogenized in an ice-cold lysis buffer containing 50 mM Tris (pH 7.5), 150 mM NaCl, 5 mM EDTA, 1% Nonidet P-40, and protease inhibitors, and then centrifuged at 13,500 × g for 15 min at 4 ˚C, as previously described [41]. The resulting supernatant was analyzed for phospho- and total PKCι levels by Western blot analysis.

For co-IP assays, tissues were homogenized in the ice-cold IP lysis buffer containing 10 mM Tris (pH 7.6), 10% glycerol, 1% Nonidet P-40, 5 mM EDTA, 5 mM EGTA, and protease inhibitors (Roche Diagnostics). After centrifugation at 13,500 × g for 30 min at 4 ˚C, the resulting supernatant extracts were incubated with a mouse anti-SORLA antibody (BD Biosciences) overnight at 4 °C, followed by a 4-h incubation with 30 μl protein G bead slurry at 4 °C. The total soluble input and bound proteins were then eluted in SDS sample buffer and subjected to Western blot analysis using antibodies against SORLA (rabbit, Protein Tech), VPS35 (goat, Origene) and βARR2 ([42], gift from Dr. Jeffrey Benovic at Thomas Jefferson University). Quantification of VPS35 and βARR2 in the IP complex was normalized to their corresponding levels in the IP buffer-soluble input and to the amount of IP’ed SORLA.

### Human Aβ40 and Aβ42 ELISA

The carbonate soluble fraction of cortical lysates was prepared from AppKI mice treated with vehicle or auranofin as previously described [43]. In brief, cortical tissues were homogenized in carbonate buffer (100 mM Na_2_CO_3_, 50 mM NaCl, pH 11.5, with inhibitors) and centrifuged at 14,000 rpm for 30 min at 4 °C. The supernatant was collected and neutralized with 0.5 M Tris–HCl (pH 6.8) at 1/10 the volume of the supernatant. The resulting lysates were then diluted five times with the assay buffer and human Aβ40 and Aβ42 levels were measured using specific ELISA kits (Invitrogen, Life Technologies), following the manufacturer’s instructions.

To measure Aβ40 and Aβ42 in the culture medium of human iPSC-derived cortical neurons, conditioned media from vehicle- or auranofin-treated cells were collected after 72 h and diluted with the assay buffer before ELISA measurement.

### Differentiation and treatment of human iPSC-derived cortical neurons

Human iPSC-derived excitatory neurons from healthy control (line EX-SeV-CW50065) and AD subjects (line EX-SeV-CW50114 and EX-SeV-CW50137) were obtained from Elixirgen Scientific (USA) and cultured following the manufacturer’s guide. 1 × 10^5^ cells were seeded per well in a 24-well plate coated with 0.002% (in PBS) poly-L-Ornithine solution and 10ug/ml laminin. Cells were treated with auranofin (0.4 µM and 1 µM for EX-SeV-CW50065; 1 µM for EX-SeV-CW50114 and EX-SeV-CW50137) for 72 h from 14 days in vitro (DIV) to 17 DIV. To knock down *SORL1* expression, DIV14 neurons were transfected with siRNAs (IDT) (5 pmol/well) against *SORL1* (hs.Ri.SORL1.13.2 and hs.Ri.SORL1.13.4) or scrambled siRNA using Lipofectamine™ RNAiMAX (Invitrogen) and then treated with auranofin or vehicle immediately after transfection. After 72 h, the culture medium was collected and tested for human Aβ40 and Aβ42 using a specific ELISA kit for each Aβ species (Invitrogen). Cells were also subjected to immunostaining and Western blot using a SORLA antibody.

### Cell culture and transfection

Neuro2A cells were grown in a 1:1 mixture of DMEM and Opti-MEM, supplemented with 5% fetal bovine serum, 100 U/ml penicillin, and 100 µg/ml streptomycin. Neuro2A cells stably expressing SNAP-tagged SORLA were generated by transfecting cells with the pSNAPf-SORLA construct, followed by selection with G418. HEK293T cells were maintained in DMEM with 10% fetal bovine serum, 100 U/ml penicillin (Fisher, #15–140–163), and 100 µg/ml streptomycin (Fisher, #15–140–163) [32]. Mycoplasma contamination was routinely checked using a detection kit. For plasmid and siRNA transfections, cells were transfected using Lipofectamine 2000 (Fisher, #11,668,027) according to the manufacturer's instructions.

Primary culture of cortical neurons from WT and *Arrb2*^*−/−*^ mice was performed as described previously [44]. Cortices from WT and *Arrb2*^*−/−*^ newborn (postnatal day 0) mice were dissected out, minced, and digested with papain (Worthington-biochem, #LK003178) for 15 min at 37 °C. Neurons (2.5 × 10^4^ cells/well) were plated onto 12 mm coverslips coated with 0.1 mg/ml poly-D-lysine (P6407, Sigma-Aldrich) in 24-well plates with Neurobasal-A medium (Fisher, #10,888,022) supplemented with 5% FBS, 2% B27, (Fisher, #17,504,044) 2% glutamax (Fisher, #35,050,061) and 0.2% gentamycin (Fisher, #15,750,060). On the second day, the medium was replaced with the feeding medium containing Neurobasal-A supplemented with 2% B27, 2% glutamax, and 0.2% gentamycin. Immunofluorescence staining was performed on DIV14 neurons.

### Immunocytochemistry and quantification of colocalization

Cells grown on coverslips were washed once with cold Dulbecco's phosphate-buffered saline (DPBS) supplemented with CaCl₂ and MgCl₂, and fixed with 4% paraformaldehyde (PFA) in PBS for 10 min on ice. Following fixation, cells were permeabilized with 0.1% Triton X-100 in PBS (PBST) for 15 min at room temperature. To block non-specific binding, cells were incubated for 1 h in blocking buffer containing 5% heat-inactivated goat serum (Thermo Fisher Scientific) in PBST. After blocking, coverslips were incubated overnight at 4 °C with specific primary antibodies (listed in Table S1), diluted in blocking buffer. After three washes with PBST, cells were incubated with Alexa Fluor®-conjugated secondary antibodies (Life Technologies) for 1 h at room temperature. Nuclei were counterstained with DAPI, and samples were mounted using ProLong™ Gold Antifade Mountant with DAPI (Life Technologies). Images were captured using Nikon A1R microscope equipped with a 60 × or 100 × oil immersion lens (1.4 NA).

Colocalization of two proteins in cultured cells was analyzed using ImageJ (v1.5i). Images were preprocessed by converting them to 8-bit format and performing background subtraction to remove nonspecific staining. Thresholding was then applied independently to each channel to accurately define positive signals. Merged composite images were generated to visualize both proteins simultaneously. Colocalization was quantified using the Coloc2 plugin in ImageJ, which calculates the Pearson’s correlation coefficient (PCC). PCC values range from –1 (complete exclusion) to + 1 (perfect colocalization), with 0 indicating no correlation. For analysis, a PCC value greater than 0.5 was considered indicative of moderate to strong colocalization, while a PCC value less than 0.5 suggested weak or partial colocalization. Changes in colocalization between groups were evaluated by calculating the fold change, defined as the PCC of the experimental group divided by the PCC of the control group. All image analyses were conducted in a blinded manner to minimize bias.

### Immunofluorescence staining of brain tissues

For brain tissue immunostaining, mice were transcardially perfused with cold PBS followed by chilled 4% PFA in 0.1 M phosphate buffer (pH 7.2), and brains were subsequently paraffin-embedded. Serial coronal Sects. (10-µm thickness) were rehydrated, permeabilized with 0.1% Triton X-100, and blocked with 5% serum in TBST (TBS containing 0.1% Tween 20) for 1 h at room temperature. Sections were then incubated overnight at 4 °C in a humidified chamber with appropriate antibodies as listed in Table S1. Following three washes with TBST, sections were incubated with Alexa Fluor®-conjugated secondary antibodies for 1 h at room temperature, washed, counterstained with DAPI, and mounted. Digital images were acquired using a Nikon A1R microscope under identical acquisition settings to ensure consistency across samples. All image analyses were conducted in a blinded manner to minimize bias.

The density of GFAP-positive astrocytes and IBA1-positive microglia was quantified in cortical and hippocampal regions using ImageJ. Background subtraction was performed to eliminate nonspecific signals, and images were converted to grayscale. Thresholding was then applied to accurately identify GFAP- or IBA1-positive cells. Regions of interest (ROIs) were defined based on the DAPI channel. The density of GFAP- and IBA1-positive cells was calculated as the percentage of the area occupied by positive staining relative to the total tissue area in each image. Quantification of Aβ deposition was also performed using ImageJ by applying thresholding to the images and calculating the percentage of the area covered by Aβ immunoreactivity. For each animal, at least 5–6 sections were quantified, and the average of these sections was presented as a single data point for that animal.

### Liquid Chromatography-Tandem Mass Spectrometry (LC–MS/MS) analysis

HEK293 cells grown on two 100 mm cell culture plates were co-transfected with a plasmid expressing Myc-tagged hSORLA and a second plasmid expressing one of two different forms of constitutively active PKCι/λ. An empty vector (pcDNA3.1) was co-transfected as the second plasmid to serve as a negative control. Forty-eight hours post-transfection, cells were treated with 50 nM Calyculin A (Thermo Scientific) for 15 min and subjected to immunoprecipitation (IP) using anti-c-Myc magnetic beads (Thermo Scientific #88,842). After thorough washing, IP’ed samples were submitted to the Augusta University Proteomics Core Facility for mass spectrometry analysis. Bead samples were incubated at 37 °C for one hour in 8 M urea denaturation buffer (50 mM Tris–HCl, pH 8) to allow IP’ed proteins to be eluted. The supernatant was then reduced with 10 mM dithiothreitol, alkylated using 55 mM iodoacetamide, and digested overnight in 50 mM ammonium bicarbonate buffer using trypsin (Thermo Scientific #90,057) at 37 °C. Digested peptides were cleaned using a C18 spin column (Harvard Apparatus #744,101) and lyophilized. Peptide samples were analyzed on an Orbitrap Fusion Tribrid mass spectrometer (Thermo Scientific) coupled with an Ultimate 3000 nano-UPLC system (Thermo Scientific). Six microliters of reconstituted peptides were first trapped and washed on a PepMap100 C18 trap (5 µm, 0.3 × 5 mm) at 20 µL/min using 2% acetonitrile in water (with 0.1% formic acid) for 10 min. Peptides were then separated on a PepMap 100 RSLC C18 column (2.0 µm, 75-µm × 150-mm) using a gradient of 2% to 40% acetonitrile with 0.1% formic acid over 120 min at a flow rate of 300 nL/min and a column temperature of 40 °C. Samples were analyzed using data-dependent acquisition (DDA) in positive mode, with the Orbitrap MS analyzer performing precursor scans at 120,000 FWHM from 400 to 1600 m/z, using quadrupole isolation. MS/MS scans were performed in top-speed mode (3-s cycle time) with an ion-trap MS analyzer and dynamic exclusion settings (repeat count of 1 and exclusion duration of 15 s). Higher-energy collisional dissociation (HCD) was used as the fragmentation method, with a normalized collision energy of 32%.

Raw MS and MS/MS spectral data were processed using Proteome Discoverer (v2.5, Thermo Scientific) and submitted for SequestHT search against the SwissProt human protein database. SequestHT search parameters included a 10 ppm precursor ion tolerance and a 0.6 Da product ion tolerance, with static carbamidomethylation (+ 57.021 Da) of cysteine, dynamic phosphorylation (+ 79.966 Da) of serine, threonine, and tyrosine, oxidation (+ 15.995 Da) of methionine, deamidation (+ 0.984 Da) of glutamine and asparagine, and dynamic N-terminal acetylation (+ 42.011 Da) of proteins. The Percolator PSM validator algorithm was used to validate peptide-spectrum matches and estimate the false discovery rate, which was set to < 1% (q-value) [45].

To complement the above shotgun approach, targeted MS was performed using Parallel Reaction Monitoring (PRM). Following precipitation using the Myc-bead and sample preparation as described above, peptides were extracted to build a PRM-MS method. PRM experiments were conducted on the same LC–MS platform using identical LC elution conditions. One signature fragment, typically the most intense fragment, was selected for each candidate peptide to calculate the peak area on the extracted ion chromatogram using Skyline software (version 24.1, MacCoss Lab, WA).

### Cycloheximide chase analysis

The cycloheximide (CHX) chase analysis was performed as previously described [46]. In brief, Neuro2A cells stably expressing SNAP-tagged SORLA were transfected with indicated plasmids. 48 h post transfection, cells were treated with 50 µg/ml of CHX and collected at 0, 2, 4, 6, 8, 12, 24, and 36 h following the treatment. To reduce variability from individual transfection events, cells grown in a large dish were transfected and subsequently divided evenly into multiple wells for treatment. SORLA protein levels were assessed by Western blot analysis, quantified by normalization to tubulin, and presented as a percentage relative to the level at the 0-h time point.

### In vitro GST pull-down

In vitro GST pull-down assays were performed as previously described [32] except that the probe was labeled with biotinylated lysine instead of [^35^S] methionine. In brief, GST fusion proteins were purified with glutathione-conjugated agarose beads (Pharmacia) from BL21 bacteria. The SORLA-C probe was prepared using the In Vitro TNT™ Quick Coupled Transcription/Translation System (Promega) and the biotinylated lysine-tRNA complex (Transcend™ tRNA, Promega) following the manufacturer's instruction. Input and bound probes were separated by SDS-PAGE and detected using the Transcend™ Non-Radioactive Translation Detection System (Promega). GST fusion proteins were detected by Western blotting using a GST antibody.

### Co-immunoprecipitation

Co-IP assays were carried out as previously described [43, 47]. Briefly, cells were lysed in ice-cold IP buffer (10 mM Tris, 1% NP-40, 10% glycerol, 5 mM EDTA, 5 mM EGTA, pH 7.6, with protease inhibitors). After centrifugation at 15,000 × g for 20 min at 4 °C, soluble extracts were incubated overnight with appropriate antibodies, followed by incubation with the Thermo Scientific™ Pierce™ Protein G Plus Agarose (Thermo Scientific™, #22,851) for an additional 4 h at 4 °C. The bound proteins were eluted in 2X SDS sample buffer (Thermo Scientific, #LC2676).

To perform Co-IP of endogenous SORLA from brain tissues, mouse cortices were dissected and homogenized on ice using an ice-cold IP buffer containing 10 mM Tris (pH 7.6), 10% glycerol, 0.5% Nonidet P-40, 5 mM EDTA, 5 mM EGTA, and protease inhibitors (Roche Diagnostics). The homogenates were rotated for 20 min at 4 °C, then centrifuged at 15,000 × g for 30 min at 4 °C. The supernatants were incubated overnight at 4 °C with an anti-SORLA antibody. The following day, the lysates were treated with 50 µl of prewashed protein G beads and incubated for 4 h at 4 °C. The beads were washed three times with lysis buffer for 10 min at 4 °C, and the bound proteins were eluted in 2X SDS sample buffer.

Proteins isolated in the IP complex and the IP buffer-solubilized input were analyzed by quantitative Western blotting. For quantification and comparison, the level of co-isolated protein was normalized to its corresponding input level and to the amount of target protein IP’ed by the antibody.

### Immunoblotting

Cell and tissue lysates were separated using SDS-PAGE and transferred to a PVDF membrane (Biorad). The membranes were blocked with 5% milk in 0.1% Tween-20 in TBS (TBS-T) for 1 h at room temperature and then incubated overnight at 4 °C with the appropriate primary antibodies (listed in Table S1) diluted in 5% BSA in TBS-T. The following day, the membranes were thoroughly washed with TBST and then probed with either fluorescence- or HRP-conjugated secondary antibodies. Membranes were scanned with the LI-COR Odyssey system, and images were analyzed using Li-COR Image Studio software (Li-COR Biosciences). Protein bands were measured in triplicate for accuracy, with GAPDH as loading controls. The data are presented as relative units to reflect protein expression levels, normalized to control values.

### Statistical analysis

During the behavioral testing and molecular analyses of this study, the experimenters were blinded to the genotypes and treatments. Data are presented as mean ± SEM, with individual points shown in column or bar graphs. Animals were excluded based solely on poor health, as assessed by veterinarians and routine colony health checks. The study employed a randomized design to minimize bias and ensure data integrity, with sample sizes determined by power analysis (80% power, 0.05 alpha) using G*Power based on rodent behavioral data. Statistical analyses were conducted using GraphPad Prism, with comparisons made using Student’s *t*-test (for two groups), one-way ANOVA (for three or more groups with one variable), or two-way ANOVA (with multi variables), as appropriate. Post hoc Tukey’s or Sidak's tests followed ANOVA where necessary. Statistical significance was set at *P* < 0.05.

## Results

### βARR2 directly interacts with SORLA, impeding the SORLA-retromer interaction

**Fig. 1:**
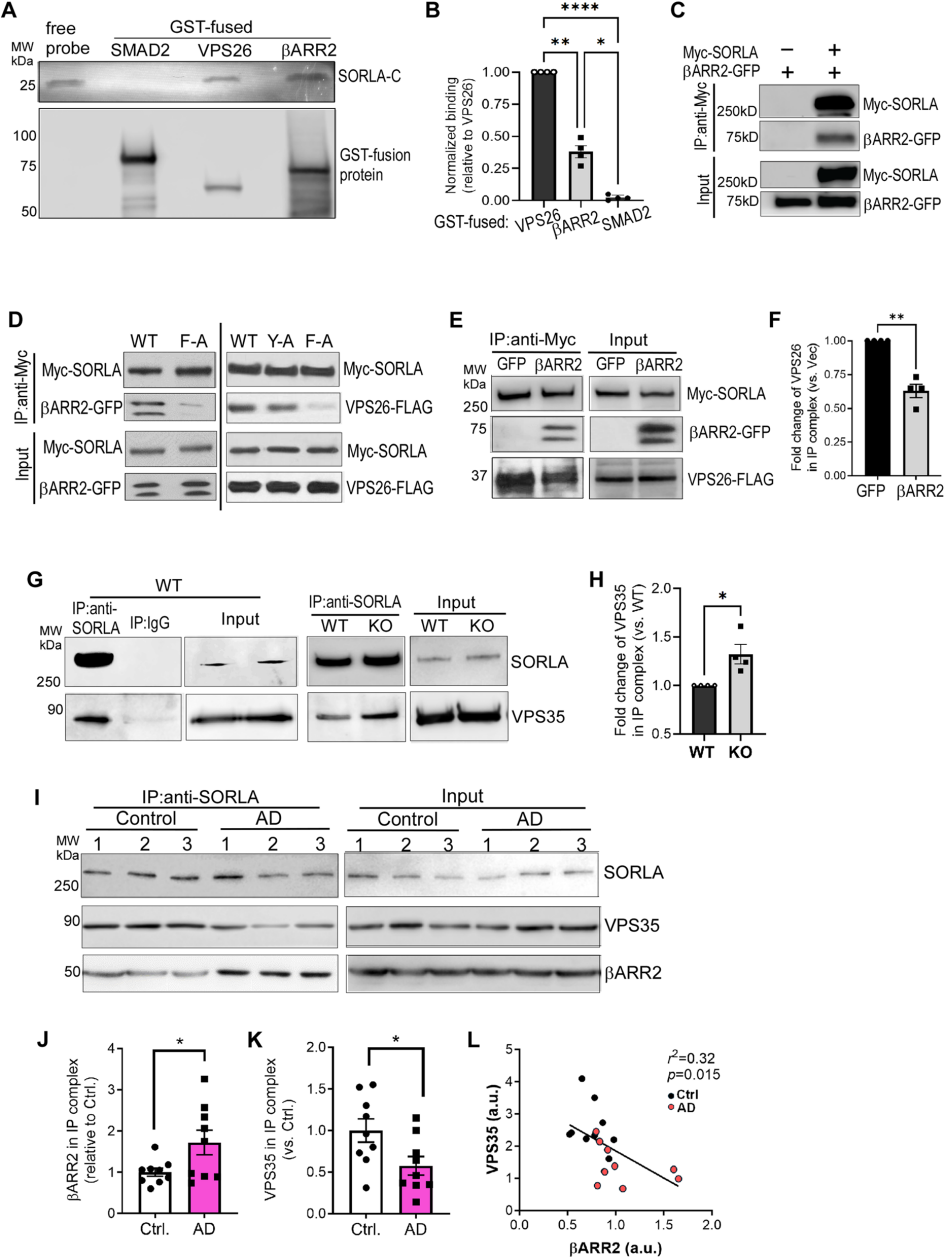
βARR2 directly interacts with SORLA and competes for the retromer interaction with SORLA. **A** Direct interaction of SORLA with βARR2 and VPS26 detected by in vitro GST pull-down assays. GST-fused βARR2, VPS26 and SMAD2 (negative control) were incubated with in vitro translated biotin-labeled SORLA-C. *Top*, Colorimetric detection of SORLA-C. Free probe, 1/50 of input. *Bottom*, Western blot with a GST-antibody showing GST-fusion proteins. **B** Quantification of the amount of SORLA-C in the pull-down complexes with indicated the GST-fusion proteins, normalized to the amount of each GST-fusion protein in the reaction. The data are presented as fold differences relative to the amount of SORLA-C bound to GST-VPS26, which was set to 1.0. *, *p* < 0.05; **, *p* < 0.01; ****, *p* < 0.0001, by one-way ANOVA Tukey’s multiple comparisons test. **C** βARR2 and SORLA form a complex in intact cells. HEK293 cells expressing βARR2-GFP alone or together with Myc-SORLA were subjected to co-IP assays using a Myc antibody. Representative blots are shown. **D** βARR2 interacts with SORLA through the FANSHY motif. HEK293 cells co-expressing βARR2-GFP or VPS26-FLAG with WT or mutant SORLA were subjected to co-IP assays using a Myc antibody. F-A, F^2172^ → A mutation; Y-A, Y^2177^ → A mutation. **E** & **F** Expression of βARR2 reduces the SORLA-VPS26 interaction. HEK293 cells co-expressing Myc-tagged SORLA and FLAG-tagged VPS26, together with GFP-tagged βARR2 or its empty vector, were subjected to co-IP assays. Representative blots (E) and quantification of the fold change of VPS26 in the Myc-SORLA-IP complex (F) are shown. **, *p* < 0.01 by paired *t* test. **G**&**H** The interaction between SORLA and the retromer complex is enhanced in the brain of βARR2 deficient mice compared to WT mice. Cortical lysates prepared from WT or *Arrb2*^*−/−*^ (KO) mice were subjected to co-IP assays using a SORLA antibody. Representative blots (G) and quantification of the fold change in VPS35 levels within the SORLA IP complex (H) are shown. Quantification was normalized to the level of VPS35 in the IP buffer-soluble input and to the amount of IP’ed SORLA. *, *p* < 0.05 by paired *t* test. **I**, **L** βARR2 and the retromer complex reciprocally interact with SORLA. Lysates prepared from control and AD postmortem PFC were subjected to co-IP assays using a mouse SORLA antibody (BD Biosciences). Representative plots (I) and quantification of the relative levels of VPS35 (J) and βARR2 (K) in the SORLA-IP complex were shown. The amount of VPS35 in the SORLA-IP complex inversely correlates with that of βARR2 (L). *, *p* < 0.05 by unpaired *t* test. All data are mean ± SEM

SORLA directly interacts with the retromer subunit, VPS26, an α-arrestin-like protein [13]. The shared structural homology between VPS26 and β-arrestins [48] motivated us to test whether βARR2 could also bind to SORLA. In in vitro GST pull-down assays, the probe containing the C-terminus of SORLA (SORLA-C) was pulled down by GST-fused βARR2 and VPS26, but not by GST-SMAD2 (an unrelated protein serving as a negative control) (Fig. 1A). The amount of SORLA-C pulled down with GST-βARR2 was significantly lower than that with GST-VPS26 (Fig. 1B). These data suggest that βARR2 directly interacts with SORLA, but with a lower affinity compared to VPS26. We then examined the βARR2-SORLA interaction in intact cells. By co-IP assays, we detected the complex formation between βARR2 and SORLA in cells co-expressing these two proteins (Fig. 1C). Additionally, an F^2172^ to A mutation at the VPS26-binding site of SORLA, ^2172^FANSHY^2177^ [13], nearly abolished its interaction with βARR2 (Fig. 1D). This mutation also drastically reduced SORLA binding with VPS26 (Fig. 1D), suggesting that SORLA binds to βARR2 and VPS26 through a similar mode.

We next sought to investigate whether βARR2 could compete for VPS26 interaction with SORLA. We found that βARR2 overexpression in cells significantly reduced the level of VPS26 co-immunoisolated with SORLA (Fig. 1E and F). The complex formation between SORLA and another subunit of retromer complex, VPS35, was also reduced in cells overexpressing βARR2 compared to control cells (Fig. S1). On the other hand, in mice lacking βARR2 expression, the complex formation between endogenous SORLA and VPS35 in the brain was significantly enhanced (Fig. 1G and H). These data suggest that βARR2 competes with the retromer complex for interacting with SORLA. Collectively, we identified a novel direct interaction between SORLA and βARR2, which competes for retromer interactions with SORLA.

We further explored whether the interaction between SORLA and βARR2 occurs in the human brain and if it is altered in AD. As shown in Fig. 1I, βARR2 and VPS35 were readily detected in the IP complex pulled down by a SORLA antibody. In AD brains, the level of βARR2 within the SORLA complex was significantly higher compared to controls (Fig. 1I and J). Conversely, the amount of VPS35 in the complex with SORLA was significantly lower in AD brains than in controls (Fig. 1I and K). Additionally, there was a negative correlation between the quantities of VPS35 and βARR2 in the complex with SORLA (Fig. 1K). These findings support the significance of the reciprocal regulation of SORLA by βARR2 and VPS proteins in the context of AD.

### βARR2 reduces SORLA localization to the TGN and promotes its redistribution to endosomes and lysosomes

**Fig. 2:**
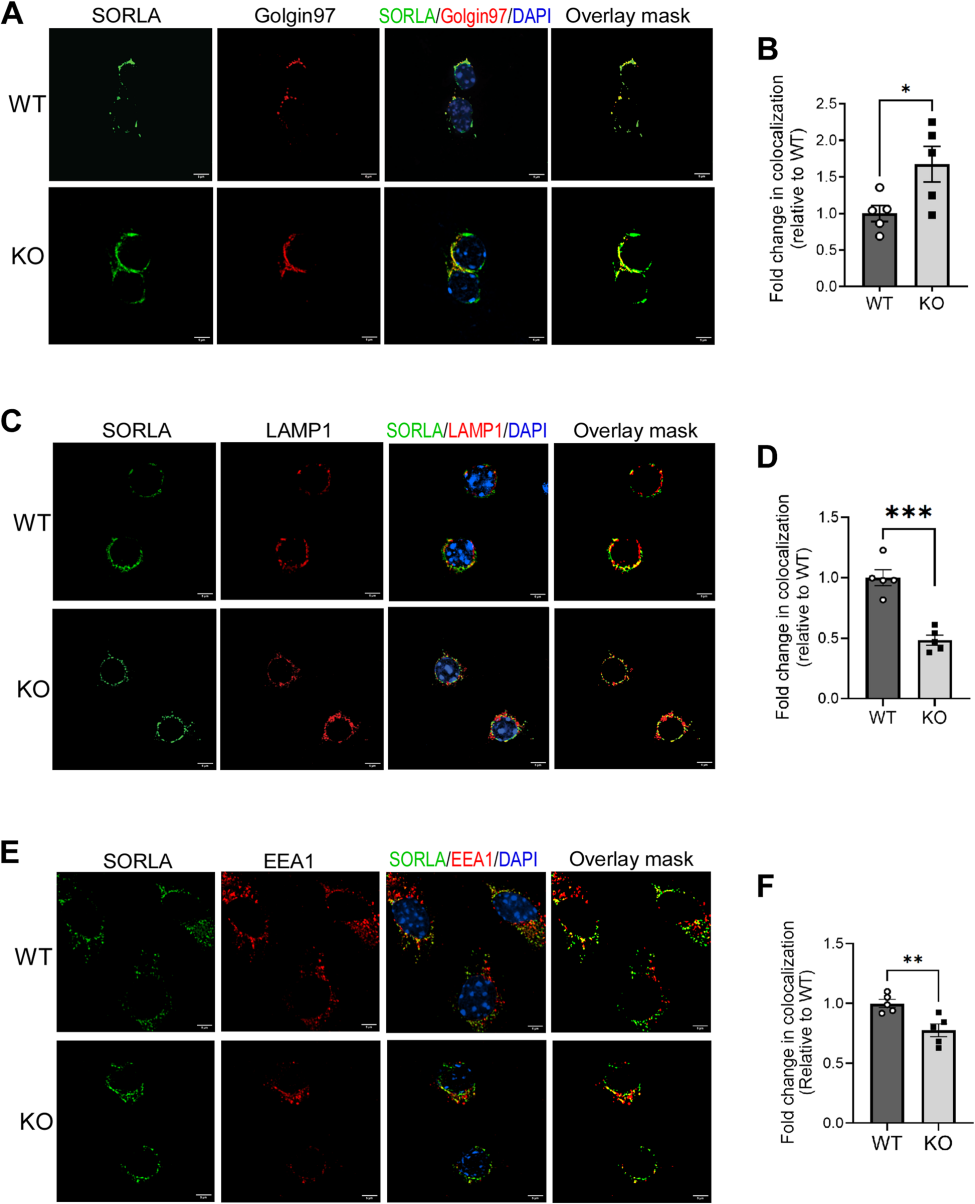
βARR2 reduces SORLA distribution to the TGN while increases its localization to endosomes and lysosomes. Primary cortical neurons (14–16 DIV) derived from WT or *Arrb2*^*−/−*^ (KO) mice were subjected to immunostaining. **A**&**B** SORLA localization to the TGN is increased in neurons lacking βARR2 expression. Representative images of SORLA and Golgin97 (A) and quantification of the colocalization coefficient between the two proteins (B) are shown. **C**&**D** SORLA localization to late endosomes/lysosomes is decreased in neurons lacking βARR2 expression. Representative images of SORLA and LAMP1 (C) and quantification of the colocalization coefficient between the two proteins (D) are shown. **E**&**F** SORLA localization to early endosomes is decreased in neurons lacking βARR2 expression. Representative images of SORLA and EEA1 (E) and quantification of the colocalization coefficient between the two proteins (F) are shown. Scale bar, 5 μm. *, *p* < 0.05, **, *p* < 0.01; ***, *p* < 0.001 by unpaired *t* test. All data are mean ± SEM

SORLA is transported to trans Golgi network (TGN) by the retromer complex [13]. Since βARR2 impedes SORLA interaction with retromers, we predicted that βARR2 would reduce SORLA trafficking to TGN. Indeed, in Neuro2A cells overexpressing βARR2, there was a significant reduction in the level of colocalization between SORLA and a TGN marker, Golgin97 (Fig. S2). Concurrently, we observed an increase in colocalization between SORLA and the late endosome/lysosome marker LAMP1 in Neuro2A cells overexpressing βARR2 (Fig. S2). By contrast, in primary neurons lacking βARR2 expression, the level of endogenous SORLA localization to the TGN was significantly increased (Fig. 2A and B), whereas its localization to late endosomes/lysosomes was significantly decreased (Fig. 2C and D), compared to that in WT neurons. These observations were made using a SORLA antibody whose specificity in immunostaining was validated using *Sorl1*^*−/−*^ brain sections (Fig. S3). SORLA localization to early endosomes (labeled by EEA1) was also decreased in *Arrb2*-deficient neurons compared to WT neurons (Fig. 2E and F). Taken together, these data suggest that βARR2 promotes redistribution of SORLA from the TGN to endosomes and lysosomes.

### βARR2 facilitates the interaction between SORLA and the ESCRT-0 complex to promote lysosomal localization and degradation of SORLA

**Fig. 3:**
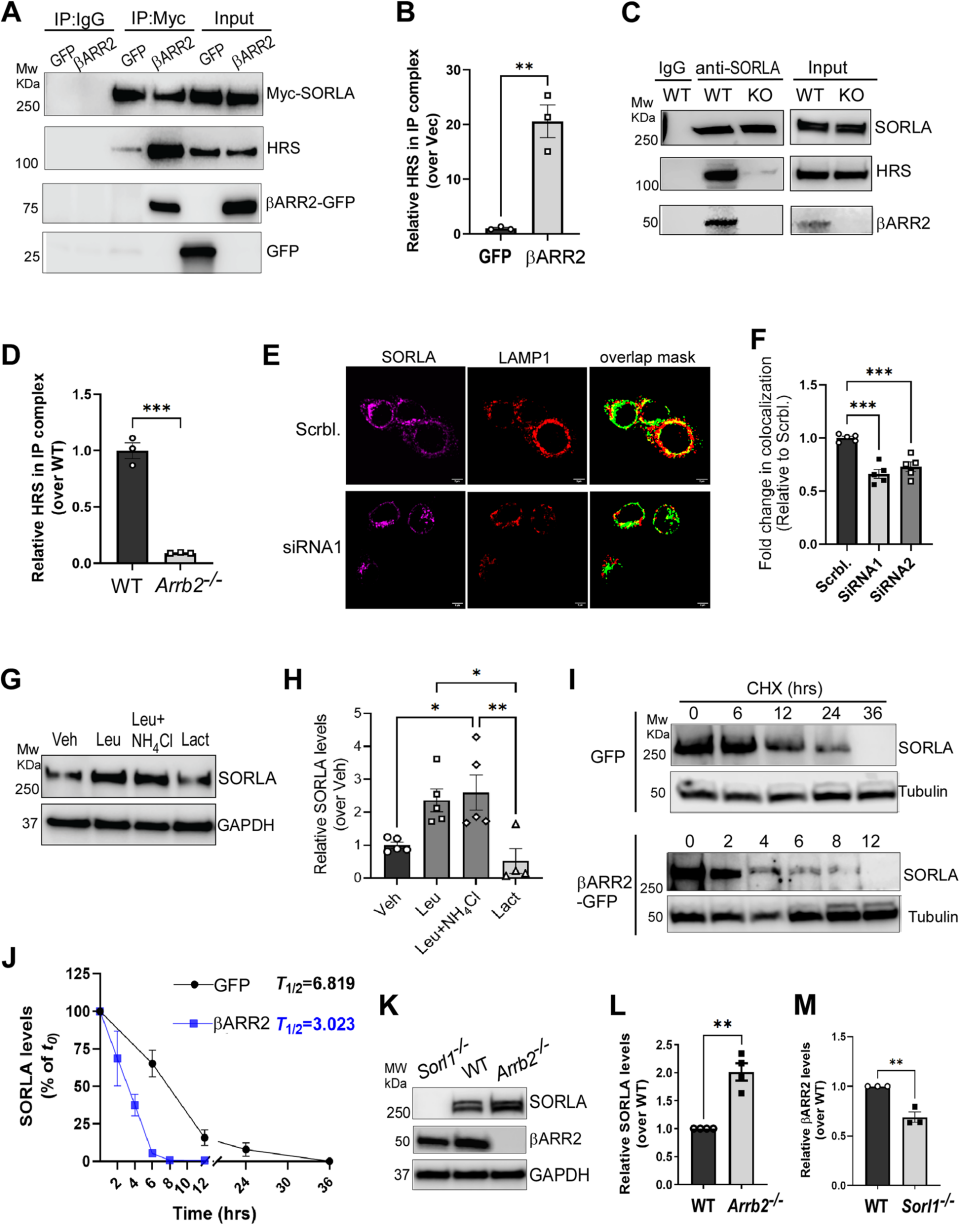
βARR2 scaffolds SORLA interaction with the ESCRT-0 complex to promote lysosome localization and degradation of SORLA. **A**&**B** Overexpression of βARR2 enhances the SORLA interaction with a key ESCRT-0 component, HRS. HEK293 cells co-expressing Myc-SORLA with βARR2-GFP, or GFP alone, were subjected to co-IP assays using a Myc antibody. Representative blots (A) and quantification of the fold change of endogenous HRS in the Myc-SORLA-IP complex (B) are shown. **, *p* < 0.01 by unpaired *t* test. **C**&**D** The interaction between SORLA and HRS is diminished in the brain of *Arrb2* deficient mice. Cortical lysates prepared from WT or *Arrb2*^*−/−*^ mice were subjected to co-IP assays using a SORLA antibody. Representative blots (C) and quantification of the fold change of HRS in the SORLA-IP complex (D) are shown. ***, *p* < 0.001 by unpaired *t* test. **E**&**F** Knocking down *Hrs* reduces SORLA localization to late endosomes/lysosomes in cells overexpressing βARR2. Neuro2A cells stably expressing SNAP-tagged SORLA were co-transfected with GFP-βARR2 and siRNAs against *Hrs* or a scrambled siRNA (Scrbl.) and then subjected to immunostaining. Representative images (E) and quantification (F) of the colocalization coefficient between SNAP-SORLA and LAMP1 are shown. Scale bar, 5 μm. ***, *p* < 0.001 by one-way ANOVA Tukey’s multiple comparisons test. **G**&**H** SORLA is degraded by lysosomes. Neuro2A cells stably expressing SNAP-tagged SORLA were treated for 24 h with vehicle, lactacystin (Lact, 10 µM), leupeptin (Leu, 50 µM), or a combination of leupeptin (Leu, 50 µM) and NH_4_Cl (20 mM). Total cell lysates were subjected to Western analysis. Representative blots (G) and quantification of the relative SORLA levels (H) are shown. *, *p* < 0.05; **, *p* < 0.01 by one-way ANOVA Tukey’s multiple comparisons test. **I**&**J** Overexpression of βARR2 reduces SORLA half-life. Neuro2A cells co-expressing SORLA with βARR2-GFP or GFP alone were subjected to cycloheximide (CHX) chase assays. Cells were harvested at indicated time points. Representative blots (I) and quantification of relative SORLA levels at different time points (J) are shown. **K-M** SORLA and βARR2 reciprocally regulate each other’s protein levels in the brain. Total cortical lysates prepared from WT, *Sorl1*^*−/−*^ or *Arrb2*^*−/−*^ mice were subjected to Western blot analysis. Representative blots (K) and quantification of relative SORLA (L) and βARR2 (M) levels in the brain are shown. **, *p* < 0.01 by unpaired *t*-test. All data are mean ± SEM

To elucidate the molecular mechanism by which βARR2 enhances the lysosome localization of SORLA, we examined whether βARR2 could facilitate the complex assembly between SORLA and the ESCRT-0 complex, which acts as the initial step in the lysosomal sorting pathway [49, 50]. In control cells, we detected a relatively weak interaction between SORLA and HRS, a subunit of the ESCRT-0 complex. This interaction was drastically increased by βARR2 overexpression (Fig. 3A and B). Additionally, we detected the interaction between SORLA and HRS in the WT mouse brain, which was nearly abolished in *Arrb2*-deficient brains (Fig. 3C and D), suggesting an essential role of βARR2 in scaffolding the assembly of the SORLA-ESCRT-0 complex. Furthermore, knockdown of *Hrs* by siRNAs in Neuro2A cells overexpressing βARR2 significantly reduced SORLA localization to late endosomes/lysosomes (Fig. 3E and F, Fig. S4), indicating a critical role of HRS and the ESCRT-0 complex in mediating the lysosomal localization of SORLA in the presence of βARR2.

We next determined whether SORLA could be degraded by lysosomes and whether βARR2 could enhance its degradation. Treatment with the lysosomal protease inhibitor leupeptin, either alone or in combination with NH_4_Cl, led to an increase in SORLA protein levels, while inhibition of proteosomes by lactacystin had a negligible effect (Fig. 3G and H), suggesting SORLA is degraded by lysosomes rather than proteosomes. Furthermore, overexpression of βARR2 markedly reduced the half-life of SORLA, as revealed by cycloheximide (CHX) chase assays (Fig. 3I and J). By contrast, SORLA protein levels were significantly higher in *Arrb2*-deficient brains compared to WT brains (Fig. 3K and L), whereas *Sorl1* mRNA levels were unaffected by genotype (Fig. S5). Intriguingly, the protein level of βARR2 was significantly lower in the brain of *Sorl1*-deficient mice compared to control mice (Fig. 3K and M), suggesting a potential reciprocal regulation of βARR2 by SORLA. Collectively, our data suggest that βARR2 facilitates the formation of the SORLA-ESCRT-0 complex to promote the lysosomal localization and degradation of SORLA.

### PKCι/λ induces SORLA phosphorylation and enhances its interaction with βARR2

**Fig. 4:**
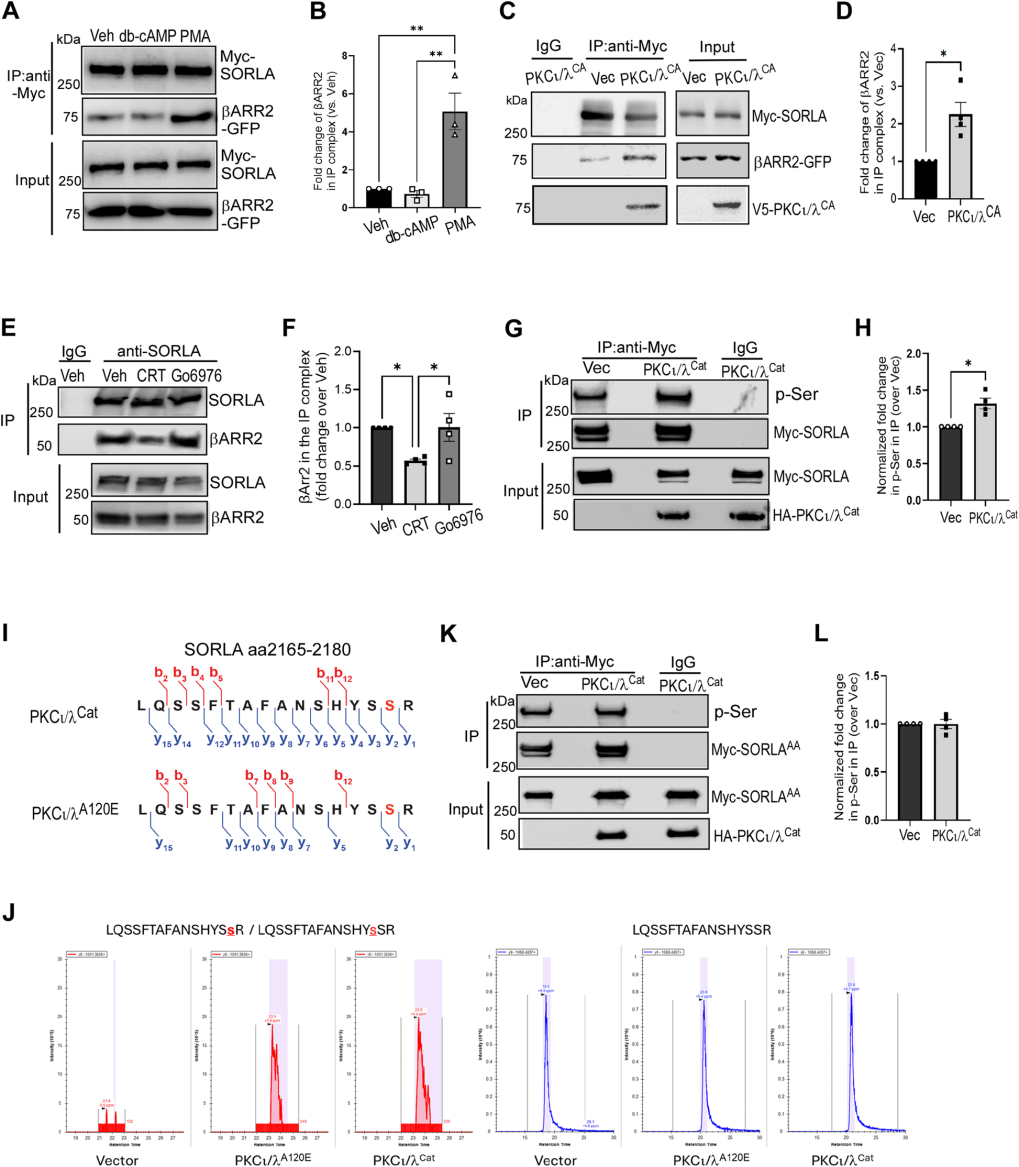
PKCι/λ induces SORLA phosphorylation and enhances its interaction with βARR2. **A**&**B** Activation of classic and novel PKC, but not PKA, enhances the SORLA-βARR2 interaction. HEK293 cells co-expressing Myc-SORLA and βARR2-GFP with indicated treatments were subjected to co-IP assays using a Myc antibody. Representative blots (A) and quantification of the fold change of βARR2 in the IP complex (B) are shown. **, *p* < 0.01 by one-way ANOVA Tukey’s multiple comparisons test. **C**&**D** Overexpression of the constitutively active PKCι/λ^A120E^ enhances the SORLA-βARR2 interaction. HEK293 cells co-expressing Myc-SORLA and βARR2-GFP together with V5-tagged PKCι/λ^CA^, or its empty vector, were subjected to co-IP assays. Representative blots (C) and quantification of the fold change of βARR2 in the IP complex (D) are shown. *, *p* < 0.05 by paired *t* test. **E**&**F** Inhibition of atypical PKC reduces the SORLA-βARR2 interaction in the mouse brain. Mice were treated with vehicle, Go6976 (5 mg/kg, i.p) or CRT0066854 (5 mg/kg, i.p.), and brain lysates were subjected to co-IP assays using a SORLA antibody. Representative blots (E) and quantification of the fold change of βARR2 in the SORLA-IP complex (F) are shown. *, *p* < 0.05 by one-way ANOVA Tukey’s multiple comparisons test. **G**&**H** Overexpression of PKCι/λ^Cat^ enhances the Ser phosphorylation level of SORLA. Neuro2A cells co-expressing Myc-SORLA with HA-tagged PKCι/λ catalytic domain (PKCι/λ^Cat^) or its empty vector were subjected to IP assays using a Myc antibody. Representative blots (G) and quantification of the fold change in phospho-Ser (p-Ser) levels of IP’ed Myc-SORLA (H) are shown. The level of p-Ser was normalized to the amount of IP’ed SORLA. *, *p* < 0.05 by paired *t* test. **I**&**J** Mass spectrometry analysis detected SORLA phosphorylation at Ser2179 in cells overexpressing PKCι/λ^Cat^ or PKCι/λ^A120E^. HEK cells were co-transfected with a plasmid encoding Myc-SORLA and a 2nd plasmid encoding HA-PKCι/λ^Cat^ or V5-PKCι/λ^A120E^. An empty vector was used as the 2nd plasmid for negative control. Prior to harvest, cells were treated with Calyculin A (50 nM, 15 min) to block phosphatase (PP1 and PP2A) activities. Cell lysates were subjected to IP using anti-Myc beads, and IP’ed samples were analyzed by mass spectrometry. The peptide sequence of interest with the b- and y-series ions detected in cell expressing PKCι/λ^Cat^ or PKCι/λ^A120E^ are shown (I). Targeted MS analysis confirmed that phosphorylation of the target peptide was detected in cells expressing constitutively active PKCι/λ, but not in control cells (J). In this analysis, we were unable to distinguish between phosphorylation at Ser2178 and Ser2179. **K**&**L** Ala mutations at Ser2178Ser2179 abolish PKCι/λ-induced increase in SORLA phosphorylation. Cells co-expressing the Myc-SORLA^AA^ mutant with HA-PKCι/λ^Cat^ or its empty vector were subjected to IP assays. Representative blots (K) and quantification of the fold change in phosphor-Ser (p-Ser) levels of IP’ed Myc-SORLA^AA^ (L) are shown. The level of p-Ser was normalized to the amount of IP’ed SORLA. All data are mean ± SEM

The FANSHY motif of SORLA, which interacts with both the retromer complex and βARR2, is flanked by putative consensus phosphorylation site for PKA and PKC [51]. Since phosphorylation-induced negative charges in partner proteins can enhance arrestin binding [52], we investigated whether manipulation of these kinases could impact the SORLA-βARR2 interaction. Cells were treated with dibutyryl-cAMP or phorbol 12-myristate 13-acetate (PMA) to activate PKA or PKC (conventional and novel isoforms). Since aPKC does not respond to PMA, we transfected cells with a constitutively active mutant form (A120E, [53]) of PKCι/λ (known as PKCι in human and PKCλ in mouse). PKCι/λ is the aPKC in the brain that is subjective to regulation, unlike PKMζ, which remains constitutively active [54]. While dibutyryl-cAMP had no effect on the SORLA-βARR2 interaction (Fig. 4A and B), both PMA treatment (Fig. 4A and B) and PKCι/λ^A120E^ expression (Fig. 4C and D) significantly enhanced it. When the endogenous activity of conventional/novel PKCs were blocked by Go6976, the SORLA-βARR2 interaction in the brain remained unchanged (Fig. 4E and F). By contrast, inhibition of aPKC activity by CRT0066854 significantly reduced the formation of the SORLA-βARR2 complex (Fig. 4E and F), suggesting a critical role of aPKC activity in promoting the SORLA-βARR2 interaction under physiological conditions. In addition, we observed PKCι/λ co-immunoisolated with SORLA in the IP complex (Fig. 4C), supporting SORLA as a direct substrate for this kinase. We therefore focused on PKCι/λ in our study.

We then tested whether the activation of PKCι/λ could enhance SORLA phosphorylation. In cells expressing the constitutively active catalytic domain of PKCι/λ (PKCι/λ^Cat^), the level of SORLA phosphorylation at Ser residues was significantly increased (Fig. 4G and H). To identify the specific intracellular phosphorylation sites targeted by PKCι/λ, we performed mass spectrometry analysis. In cells overexpressing either PKCι/λ^A120E^ or PKCι/λ^Cat^, but not in those transfected by the empty vector, phosphorylation at Ser2179 was consistently detected (Fig. 4I and Fig. S6). Phosphorylation at Ser2178 was also observed in cells overexpressing PKCι/λ^A120E^ (Fig. S6). We did not detect double phosphorylation signals simultaneously at both sites. In complementary targeted analyses, phosphorylation of the target peptide was clearly detected in cells expressing PKCι/λ^A120E^ or PKCι/λ^Cat^, but not in vector-transfected control cells (Fig. 4J). These data demonstrate that PKCι/λ activation induces SORLA phosphorylation at Ser2178 or Ser2179. Further supporting this, mutation of these Ser residues to Ala abolished the PKCι/λ^Cat^-induced increase in Ser phosphorylation (Fig. 4K and L), validating PKCι/λ-mediated phosphorylation at the Ser2178 or Ser2179 site.

### PKCι/λ promotes SORLA localization to lysosomes and reduces SORLA stability

**Fig. 5:**
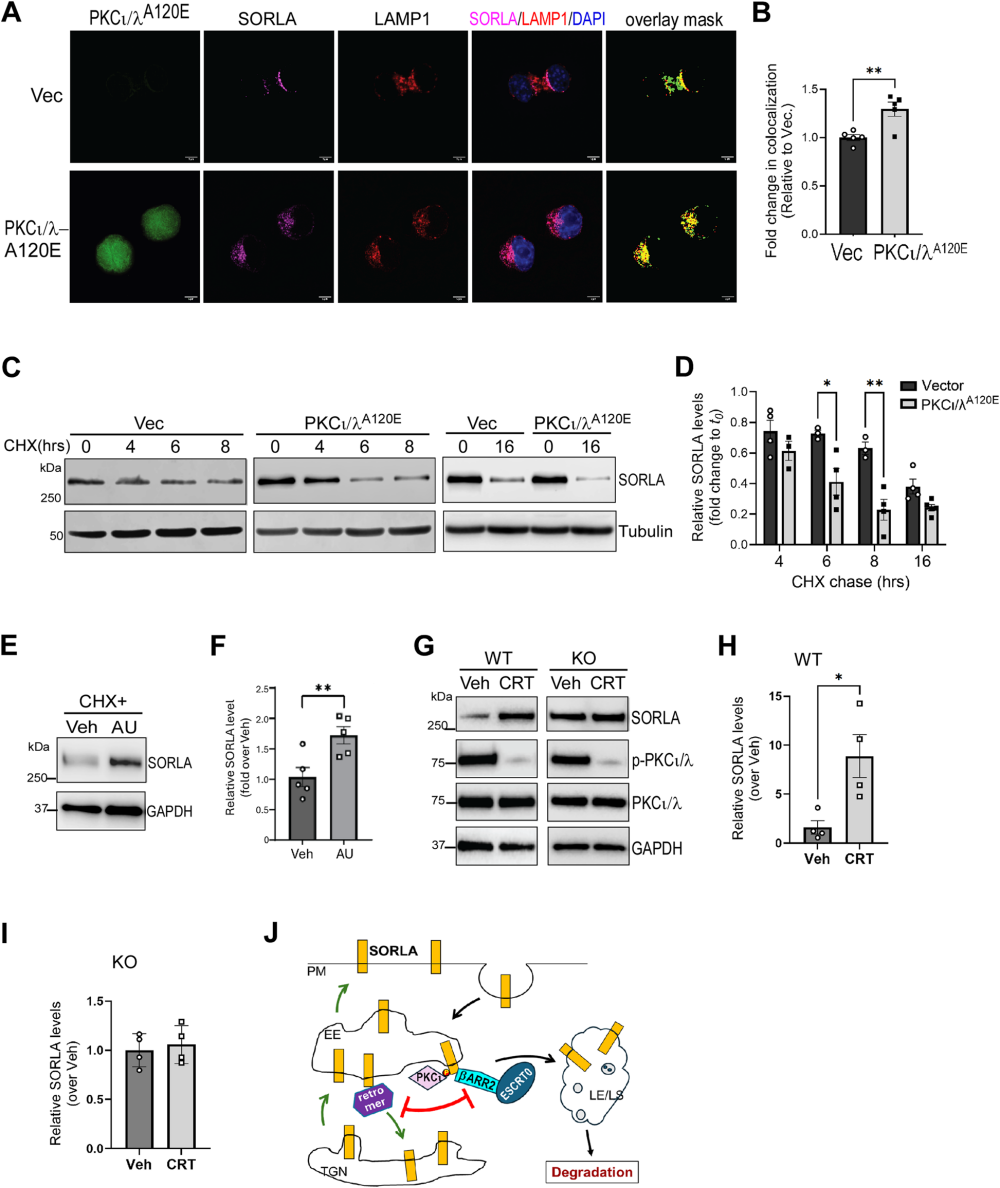
PKCι/λ activation increases SORLA localization to lysosomes and reduces its stability in a βARR2-dependent manner. **A**&**B** Overexpression of PKCι/λ^A120E^ increases SORLA localization to late endosomes/lysosomes. Neuro2A cells co-overexpressing Myc-SORLA with PKCι/λ^A120E^ or empty vector were subjected to immunocytochemistry. Representative images (A) and quantification of the colocalization coefficient between SORLA and LAMP1 (B) are shown. Scale bar, 5 μm. **, *p* < 0.01 by unpaired *t* test. **C**&**D** Overexpression of PKCι/λ^A120E^ accelerates SORLA degradation. Neuro2A cells co-overexpressing Myc-SORLA with PKCι/λ^A120E^ or the empty vector were subjected to CHX chase assays for the indicated time points. Representative blots (C) and quantification of relative SORLA levels (D) are shown. *P* < 0.0001 for the difference between PKCι/λ^A120E^ and empty vector groups (column factor) by two-way ANOVA. *, *p* < 0.05; **, *p* < 0.01 by two-way ANOVA Sadak’s multiple comparisons test. **E**&**F** Blocking PKCι/λ by auranofin increases SORLA stability. Neuro2A cells expressing Myc-SORLA were subjected to CHX chase for 16 h in the presence of vehicle or auranofin (AU). Representative blots (E) and quantification of relative SORLA levels (F) are shown. **, *p* < 0.01 by unpaired t test. **G**-**I** In vivo blocking aPKC increases SORLA levels in the brain in WT, but not βARR2 deficient, mice. WT or *Arrb2*^*−/−*^ (KO) mice were treated with an aPKC blocker, CRT0066854 (i.p., 5 mg/kg, twice daily for 2 weeks). Total cortical lysates were subjected to Western analysis. Representative blots (G) and quantification of relative SORLA levels in WT (H) and KO (I) mice are shown. The level of SORLA was normalized to GAPDH and represented as fold change relative to vehicle control (set as 1.0). *, *p* < 0.05 by unpaired *t* test. **J** Schematic illustration of the regulation of SORLA trafficking and degradation by the PKCι/λ-βARR2 axis. PM, plasma membrane; EE, early endosome; TGN, trans Golgi network; LE/LS, late endosome/lysosome

Given that PKCι/λ activation enhances the SORLA-βARR2 interaction and that βARR2 promotes the lysosomal localization and degradation of SORLA, we predicted that PKCι/λ activation would increase the lysosomal localization and degradation of SORLA. Indeed, in cells overexpressing constitutively active PKCι/λ^A120E^, the colocalization of SORLA with LAMP1 was significantly increased (Fig. 5A and B). Concurrently, SORLA localization in the TGN was significantly reduced (Fig. S7A and B). Furthermore, overexpression of PKCι/λ^A120E^ accelerated the degradation of SORLA, as revealed by CHX chase assays (Fig. 5C and D). By contrast, blocking PKCι/λ by auranofin significantly increased SORLA stability (Fig. 5E and F). Moreover, in vivo inhibition of PKCι/λ by CRT0066854, (i.p., 5 mg/kg, twice daily for 2 weeks) significantly increased the SORLA level in the brain of WT mice, but not in mice lacking βARR2 expression (Fig. 5G-I, Fig. S7C), suggesting that βARR2 is required for PKCι/λ-mediated regulation of SORLA stability. Taken together, our data collectively suggest that PKCι/λ promotes the lysosomal localization and degradation of SORLA by enhancing the SORLA-βARR2 interaction.

### Inhibition of PKCι/λ by auranofin increases SORLA levels, attenuates the SORLA-βARR2 interaction, and reduces amyloidogenic processing of APP in AD model mice

**Fig. 6:**
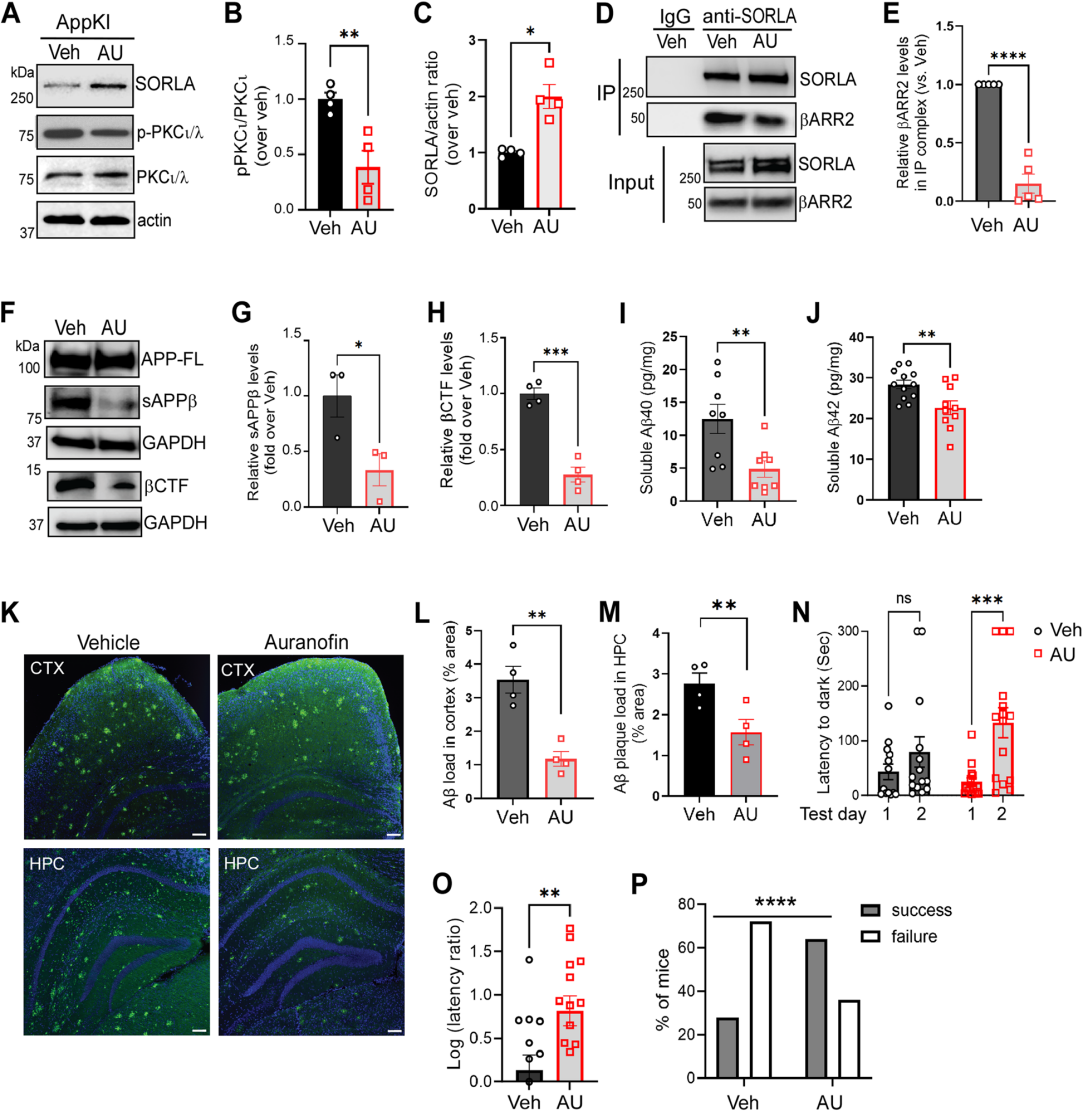
Blocking PKCι/λ by auranofin increases SORLA levels, attenuates the SORLA-βARR2 interaction, reduces amyloidogenic processing of APP, and improves cognition in AppKI mice. AppKI mice were treated with vehicle (Veh) or auranofin (AU, 5 mg/kg, oral gavage, once daily) for 7 weeks beginning at 15 weeks of age. **A**-**C** Auranofin treatment reduces the PKCι/λ activity and increases the SORLA level. Cortical lysates prepared from AppKI mice with indicated treatment were subjected to Western blot analysis. Representative blots (A) and quantification of the relative levels of phospho-PKCι/λ (p-PKCι/λ, B) and SORLA (C) are shown. *, *p* < 0.05; **, *p* < 0.01 by unpaired *t* test. **D**&**E** Auranofin treatment reduces the SORLA-βARR2 interaction. Cortical lysates from mice with indicated treatment were subjected to IP assays using a SORLA antibody. Representative blots (D) and quantification of the relative level of βARR2 in the IP complex (E) are shown. The level of βARR2 in the IP complex was normalized to the level of βARR2 in the IP buffer-soluble input and to the amount of IP’ed SORLA. ****, *p* < 0.0001 by paired *t* test. **F**–**H** BACE1 cleavage of APP is reduced in auranofin-treated AppKI mice. Representative blots (D) and quantification of the relative levels of sAPPβ (E) and βCTF (F) are shown. *, *p* < 0.05; ***, *p* < 0.001 by unpaired *t* test. **I**&**J** auranofin treatment reduces the levels of soluble human Aβ40 and Aβ42 in the cortex. The carbonate soluble fraction of cortical lysates prepared from mice with indicated treatments were subjected to ELISA. **, *p* < 0.01 by unpaired *t* test. **K**, **M** Auranofin-treatment reduces Aβ deposition in AppKI mice. Brain slices from AppKI mice with indicated treatment were subjected to immunostaining using an Aβ antibody. Representative images (K), quantification of Aβ load in the cortex (L) and hippocampus (M) are shown. Scale bar, 100 μm. *, *p* < 0.05; **, *p* < 0.01 by unpaired *t*-test. **N**-**P** Auranofin treatment improves cognitive performance in AppKI mice. Mice with indicated treatment were assessed in passive avoidance tests. Quantification of latency to dark on training (Day 1) and test (Day 2) days (N), the Log(Day2/Day1 latency ratio, O), and the percentage of mice showing successful avoidance (Day2/Day1 latency ratio > 3, P) are shown. ***, *p* < 0.001 by two-way ANOVA Sadak’s multiple comparisons in N. **, *p* < 0.01 by unpaired *t* test in O. ****, *p* < 0.0001 by Fisher’s exact test in P

Encouraged by our mechanistic studies demonstrating the critical role of the PKCι/λ-βARR2 axis in disrupting the SORLA-retromer interaction and promoting SORLA lysosomal trafficking and degradation (illustrated in Fig. 5J), we sought to determine whether inhibiting this kinase could increase SORLA levels and subsequently reduce Aβ pathology and cognitive deficits in an AD mouse model, *App*^*NL−G−F/NL−G−F*^ (referred to as AppKI) mice. The PKCι/λ inhibitor auranofin (5 mg/kg/day) was administered via oral gavage for 7 weeks, starting at 15 weeks of age, when Aβ pathology begins to emerge in these mice [36]. Notably, daily oral administration of auranofin at an even lower dose (2 mg/kg/day) has been shown to achieve low micromolar concentrations in the mouse brain [55]. As expected, auranofin treatment resulted in a significant reduction in PKCι/λ activity compared to vehicle-treated controls, evidenced by a decrease in phospho-PKCι/λ levels at the activation loop (Thr403, Fig. 6A and B). Concurrently, SORLA protein levels were significantly elevated in the brain of auranofin-treated mice compared to controls (Fig. 6A and C). Moreover, auranofin treatment significantly reduced the complex formation between SORLA and βARR2 in the brain (Fig. 6D and E), further supporting the role of PKCι/λ in promoting the SORLA-βARR2 interaction.

Given that SORLA inhibits amyloidogenic processing of APP [8], we next investigated whether the increased SORLA levels observed in the brains of auranofin-treated mice would result in reduced BACE1-mediated cleavage of APP and decreased Aβ generation. In auranofin-treated AppKI mice, the levels of both soluble APPβ and βCTF in the brain were significantly lower than those in vehicle-treated littermates, while total APP levels were unchanged between the two groups (Fig. 6F- H). Consistently, the levels of soluble human Aβ40 (Fig. 6I) and Aβ42 (Fig. 6J) were significantly decreased in the cortex of auranofin-treated mice compared to controls. These data suggest that blocking PKCι/λ reduces amyloidogenic processing of APP by BACE1 and decreases Aβ generation.

### Auranofin treatment ameliorates Aβ-related pathology and improves cognition

We further assessed Aβ burdens and gliosis in the brain of AppKI mice treated with either auranofin or vehicle. Auranofin treatment resulted in a significant reduction in Aβ plaque load in both the cortex and hippocampus (Fig. 6K-M). Furthermore, auranofin-treated AppKI mice exhibited significantly decreased astrocytic and microglial reactivity of, as evidenced by reduced GFAP and IBA-1 (Fig. S8) immunostaining, respectively, suggesting that auranofin treatment attenuates neuroinflammation.

We evaluated behavioral outcomes in AppKI mice treated with either auranofin or vehicle. Auranofin treatment did not affect general activity or anxiety-like behaviors, as assessed by the open field and elevated zero maze tests (Fig. S9). Consistent with our previous reported [40], vehicle-treated AppKI mice displayed deficits in associative learning in the passive avoidance test (Fig. 6N). Notably, auranofin treatment rescued this behavioral deficit (Fig. 6N). The latency-to-dark ratio between the two test days (Fig. 6O), which reflects associative memory, as well as the percentage of mice showing successful avoidance (defined as a day 2/day 1 ratio ≥ 3, Fig. 6P), were significantly higher in auranofin-treated mice compared to vehicle-treated controls. These data suggest that auranofin treatment improves cognitive function in AppKI mice. Since cognitive behavioral tests were conducted five days after the final auranofin administration, it is unlikely that the observed improvements were due to acute symptomatic effects. Instead, we interpret these results as reflecting the amelioration of underlying pathological changes by auranofin treatment.

### PKCι/λ is hyperactive in AD, and auranofin reduces Aβ production in AD iPSC-derived neurons by increasing SORLA levels

**Fig. 7:**
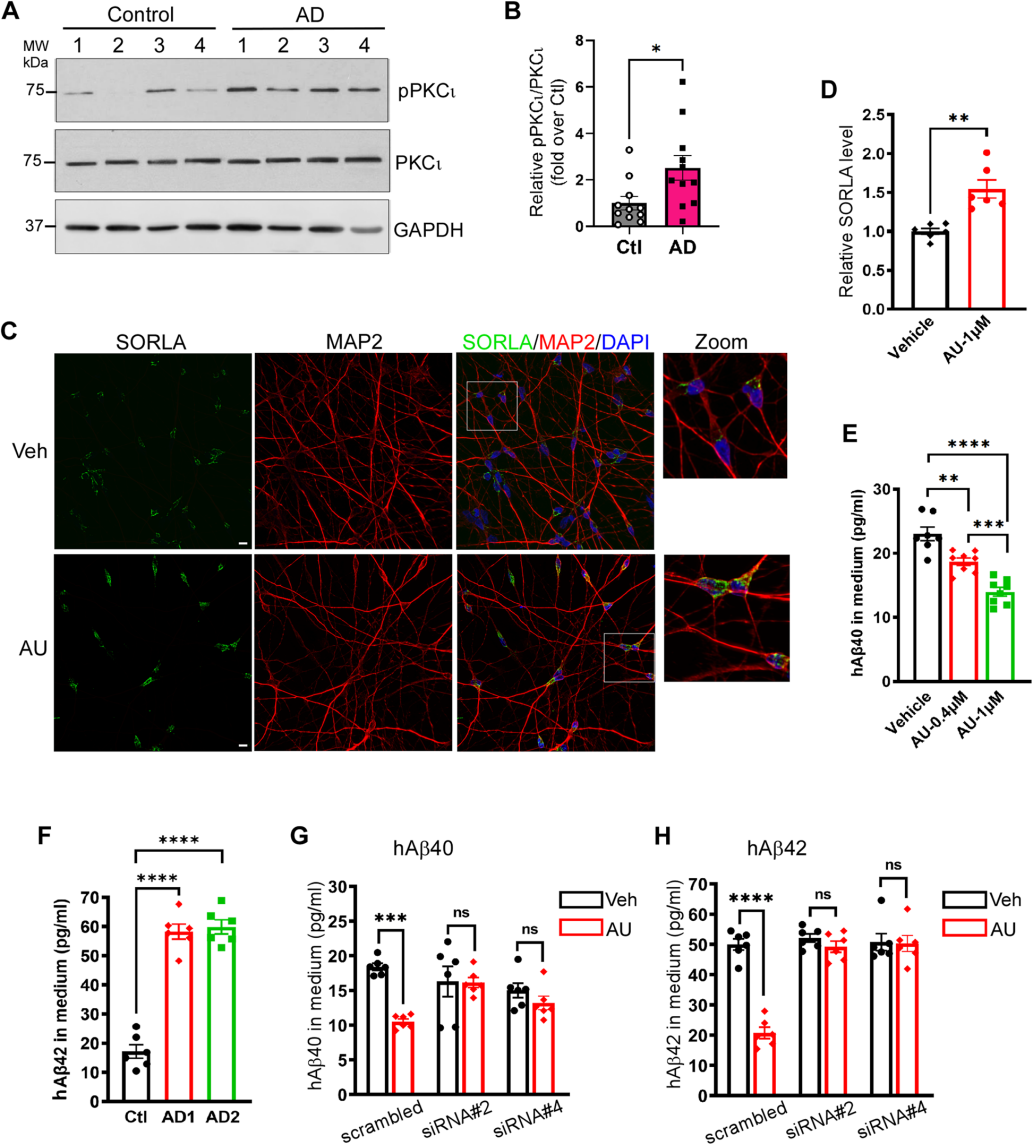
PKCι/λ is hyperactive in AD and blocking it by auranofin reduces Aβ generation through increasing SORLA levels in AD neurons. **A**&**B** The activity of PKCι/λ is increased in AD brains compared to control subjects. Total lysates of postmortem PFC from AD or control subjects were subjected to Western blot analysis. Representative blots (A) and quantification of relative levels of phospho-PKCι (pPKCι) over total PKCι (B) are shown. *, *p* < 0.05 by unpaired *t* test. **C**-**E** Auranofin treatment increases SORLA levels while reducing Aβ generation. Neurons derived from control iPSCs (line EX-SeV-CW50065, 15 DIV) were treated with auranofin at indicated concentrations for 72 h. Representative images of SORLA immunostaining (C), quantification of relative SORLA levels in cells (D), and Aβ40 concentrations in culture medium (E) are shown. Scale bar, 10 μm. **, *p* < 0.01 by unpaired *t* test in D. **, *p* < 0.01; ***, *p* < 0.001; ****, *p* < 0.0001 by one-way ANOVA Tukey’s multiple comparisons in E. **F** Neurons derived from AD iPSCs produced higher levels of Aβ42 compared to those derived from control iPSCs. ***, *p* < 0.001; ****, *p* < 0.0001 by one-way ANOVA Tukey’s multiple comparisons. **G**&**H** Auranofin reduces Aβ generation in AD neurons in a SORLA-dependent manner. Cultured neurons derived from AD iPSCs (line EX-SeV-CW50114) were transfected with siRNAs against *SORL1* and treated with auranofin for 72 h. Quantification of Aβ40 (G) and Aβ42 (H) concentrations in culture medium are shown. ***, *p* < 0.001 ****, *p* < 0.0001 by two-way ANOVA Sadak’s multiple comparisons test. Data are mean ± SEM

The above in vitro and animal studies suggest that the PKCι/λ-βARR2-SORLA axis plays a key role in regulating AD pathogenesis. We then investigated the relevance of this axis using human tissues and iPSC-derived neurons. We first examined PKCι/λ activity in human AD brains and found that the level of phospho-PKCι/λ (indicating active PKCι/λ) was significantly elevated in AD brains compared to controls (Fig. 7A and B), suggesting that PKCι/λ is hyperactive in human AD brains.

We then tested whether inhibition of PKCι/λ could increase SORLA levels in human iPSC-derived cortical neurons, similar to the effect observed in AppKI mice. Auranofin treatment for 72 h significantly increased SORLA levels in these neurons (Fig. 7C and D) and dose-dependently reduced Aβ levels (Fig. 7E). In neurons derived from two independent AD iPSC lines, more Aβ42 was produced than in normal control iPSC-derived neurons (Fig. 7F). In these AD neurons, auranofin treatment significantly reduced the levels of both Aβ40 and Aβ42 production compared to vehicle treatment (Fig. 7G and H and Fig. S10). We further addressed whether auranofin’s effects on Aβ production was due to the elevation of SORLA by this treatment. We screened and identified two siRNAs that effectively reduced SORLA expression in cells (Fig. S10) and transfected AD neurons with these siRNAs. Knocking down *SORL1* with these siRNAs abolished the effect of auranofin on Aβ production (Fig. 6G and H and Fig. S10). Collectively, our data suggest that blocking PKCι/λ with auranofin effectively reduces Aβ production in human AD neurons by elevating SORLA levels.

## Discussion

In the present study, we identified a novel interaction between SORLA and βARR2, which inhibits the interaction of SORLA with the retromer complex and disrupts its retrograde trafficking. This SORLA-βARR2 interaction, which is enhanced by PKCι/λ-mediated phosphorylation of SORLA, leads to the ESCRT-0 complex-directed localization into late endosomes/lysosomes, ultimately resulting in the degradation of SORLA (illustrated in Fig. 4J). Based on these mechanistic findings, our preclinical tests demonstrated that the PKCι/λ inhibitor auranofin significantly increases SORLA levels and reduces Aβ pathology in an AD mouse model. Additionally, auranofin treatment alleviates gliosis and improves cognitive function in AppKI model mice. Moreover, in human AD iPSC-derived neurons, auranofin treatment effectively elevates SORLA levels and decreases Aβ levels. Our study reveals a molecular mechanism by which SORLA stability is regulated and further suggests the PKCι/λ-βARR2 axis as an invaluable target for AD treatment. Notably, auranofin is an FDA-approved drug and could potentially be repurposed for AD.

SORLA interaction with the retromer complex is crucial for its retrograde trafficking [13]. Through its C-terminal FANSHY sequence, SORLA directly interacts with VPS26 [13], whose structure is closely related to β-arrestins [48]. In the current study, we identified a novel direct interaction between SORLA and βARR2, which, similar to the interaction with VPS26, occurs through the FANSHY motif within SORLA. The intrinsic binding affinity of βARR2 for SORLA is lower than that of VPS26. Overexpression of βARR2 in cells competes with VPS26 and the retromer complex for SORLA binding. Furthermore, βARR2 binding to SORLA scaffolds the interaction between SORLA and the ESCRT-0 complex, which is a key player in lysosome sorting [49, 50]. As a result, βARR2 reduces SORLA’s retrograde trafficking into TGN and promotes its localization to lysosomes for degradation. βARR2 has been shown to enhance γ secretase localization in detergent resistant membranes to promote Aβ generation downstream of GPCR activation [29]. Our study thus suggests a novel mechanism by which βARR2 regulates amyloid metabolism through directly interacting with SORLA and modulating its trafficking and stability. Both mechanisms would contribute to the previously reported reduction in Aβ generation in neurons lacking βARR2 expression [29]. SORLA has recently been found to dimerize via the fibronectin-type-III (3Fn)- and VPS10p-domains, and dimerization enhances its interaction with the retromer complex [56]. It is worth noting that βARR2 can form and function as dimers [57, 58]. Whether the interaction between SORLA and βARR2 involves dimerization of either partner remains to be investigated.

The C-terminal intracellular region of SORLA contains multiple Ser and Thr residues within putative consensus cites for several kinases, including PKA, PKC and rho activated coiled coil protein kinase 2 (ROCK2). Previous studies have shown that activation of the conventional and novel PKC isoforms and ROCK2 regulates SORLA phosphorylation and shedding in cultured cells [51, 59]. In this study, we found that pharmacological activation of conventional and novel PKC isoforms, as well as expression of a constitutively active form of aPKC, enhanced the SORLA–βARR2 interaction in Neuro2A cells. These findings align with the notion that arrestins preferentially interact with phosphorylated proteins, as the polar core of arrestins binds more effectively to the concentrated negative charges introduced by phosphorylation [52]. However, in vivo inhibition of the conventional and novel PKC isoforms had a negligible effect on the SORLA–βARR2 interaction in the mouse brain. In contrast, inhibition of aPKC disrupted the endogenous SORLA–βARR2 interaction in the brain, highlighting the critical role of aPKC in regulating this interaction under physiological conditions. We further discovered that PKCι/λ interacts with SORLA and induces phosphorylation at S2178 and S2179 sites adjacent to the FANSHY motif. Although VPS26 also contains a polar core, different from arrestins, it does not have a phosphate sensor, and the polar core does not affect its function [48]. As a result, PKCι/λ-mediated phosphorylation of SORLA favors interaction with βARR2, leading to reduced retrograde trafficking and increased lysosomal localization and degradation of SORLA.

The presence of PKCι/λ has been found in tau-positive inclusions in tauopathies [60]. In addition, PKCι/λ downstream of insulin signaling is hyperactive and increases BACE1-cleavage of APP and Aβ production in hyperinsulinemia [61]. Our study reveals a novel role of PKCι/λ in AD pathogenesis. Through enhancing the SORLA-βARR2 interaction, PKCι/λ activation promotes lysosomal localization and degradation of SORLA. Inhibition of PKCι/λ, on the other hand, increases SORLA levels in a βARR2-dependent manner. We also found elevated PKCι activity in AD, further supporting its disease relevance and potential as a therapeutic target. Indeed, our preclinical tests demonstrated that inhibition of PKCι/λ by auranofin, which targets its PB1 domain [62], significantly increases SORLA levels, disrupts the SORLA-βARR2 interaction, reduces BACE1-cleavage products and Aβ levels, and improves cognitive functions in AD model mice. Furthermore, auranofin treatment increases SORLA levels and reduces Aβ levels in human AD neurons in a SORLA-dependent manner, further supporting the blockade of PKCι/λ as an effective strategy to increase SORLA levels and mitigate AD pathogenesis.

Auranofin is an FDA-approved drug for rheumatoid arthritis and is well recognized for its effects on peripheral inflammatory pathways [63]. Additionally, auranofin has been shown to inhibit astrocyte cytotoxic secretions and protect neurons from associated toxicity [55]. We cannot rule out that these other actions may contribute to the improved pathology and cognition following auranofin treatment. Nevertheless, the fact that its effect on Aβ generation relies on SORLA, as shown in Fig. 6, strongly supports the notion that increasing SORLA stability through inhibition of PKCι/λ acts as a major, if not sole, mechanism for its role in reducing amyloidogenesis. Consistently, the effect of auranofin in reducing Aβ is significant even when treating AppKI mice at 14 months old, although at this age its impact on neuroinflammation is negligible [64]. The beneficial effects of auranofin in reducing Aβ generation in human AD neurons, as well as in alleviating AD-related pathology and cognitive deficits in AD model mice, as demonstrated in the current study, warrant a clinical trial for its immediate use in treating AD.

## Conclusions

In conclusion, our research uncovers a novel direct interaction between SORLA and another AD-related genetic factor, βARR2, and reveals how the PKCι/λ-βARR2 axis controls SORLA trafficking and stability. These findings offer mechanistic insights into the regulation of SORLA stability and shed new light on how different genetic factors can interact to impact AD pathogenesis. This study also underscores the possibility of raising SORLA levels by blocking PKCι/λ as a viable strategy to lower Aβ synthesis and ameliorate AD-related phenotypes. Notably, auranofin, a medication for rheumatoid arthritis that has received FDA approval, may potentially be repurposed for AD treatment, providing a promising and quick therapy alternative for this devastating disease.

## Supplementary Information

Supplementary Material 1.

Supplementary Material 2.

## Data Availability

All data needed to evaluate the conclusions in the paper are present in the paper and/or the Supplementary Materials.

## Abbreviations

AD: Alzheimer’s disease
aPKC: Atypical PKC
APP: Amyloid precursor protein
AppKI: *App* ^*NL*^^*−*^^*G*^^*−*^^*F/NL*^^*−*^^*G*^^*−*^^*F*^
βARR2: β-Arrestin2
CHX: Cycloheximide
Co-IP: Co-immunoprecipitation
DPBS: Dulbecco's Phosphate-Buffered Saline
iPSCs: Induced pluripotent stem cells
GPCRs: G protein-coupled receptors
GWAS: Genome-wide association studies
KI: Knock-in
KO: Knockout
PMA: Phorbol 12-myristate 13-acetate
SORLA: Sorting-related receptor with A repeat
TGN: Trans Golgi network
VPS10: Vacuolar protein sorting 10
WT: Wild-type
