# Supplementary material for: The PKCι-β-arrestin2 axis disrupts SORLA retrograde trafficking, driving its degradation and amyloid pathology in Alzheimer’s disease
Source: Mol Neurodegener. 2025 Jun 23;20:76. doi: 10.1186/s13024-025-00865-6 (PMC12186327; doi:10.1186/s13024-025-00865-6)
Supplement: Supplementary file 1 — Supplementary Material 1. [file 13024_2025_865_MOESM1_ESM.pdf]

Supplementary Information

The PKCi-β-arrestin2 axis disrupts SORLA retrograde trafficking, driving its degradation and amyloid pathology in Alzheimer's disease

Hasibur Rehman[1*], Shun Yan[2*], Shalini Saggu[1,3*], Mae Aida[1,3], Fang Zhang[3$], Yang Shu[1], Alexis Jones[1], Amy Trang[1], Emily Dew[1], Wenbo Zhi[2], Emily T. Claeboe[4], Anthony J. Baucum II[4,5], Guangyu Wu[6], Kai Jiao[2] and Qin Wang[1,3]

[1] Department of Neuroscience and Regenerative Medicine, Medical College of Georgia at Augusta University, Augusta, GA 30912.

[2] Center for Biotechnology and Genomic Medicine, Medical College of Georgia at Augusta University, Augusta, GA 30912.

[3] Department of Cell, Developmental and Integrative Biology, University of Alabama at Birmingham, Birmingham, AL 35294.

[4] Department of Pharmacology and Toxicology, Indiana University School of Medicine, Indianapolis, IN 46202.

[5] Stark Neurosciences Research Institute, Indiana University School of Medicine, Indianapolis, IN 46202.

[6] Department of Pharmacology and Toxicology, Medical College of Georgia at Augusta University, Augusta, GA 30912.

Correspondence: Qin Wang, Medical College of Georgia at Augusta University, 1120 15th Street, Augusta, GA 30912, E-mail: qiwang@augusta.edu.

[*] These authors contribute equally.

Supplementary Figures

Fig. S1

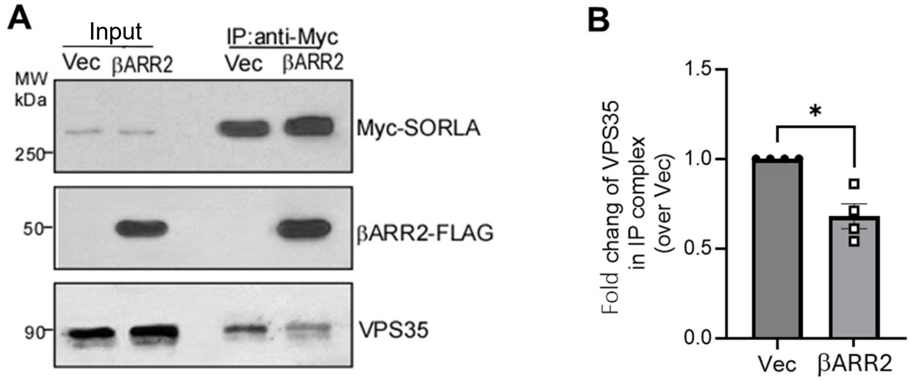

Fig. S1. Overexpression of βARR2 reduces the complex formation between SORLA and VPS35. Cells co-expressing Myc-SORLA with βARR2 or empty vector were subjected to co-IP assays using a Myc antibody. Myc-SORLA, βARR2-GFP and endogenous VPS35 were detected by Western blot analysis. Representative blots (A) and quantification of the fold change of VPS35 in the Myc-SORLA-IP complex (B) are shown. *, $p<0.05$ by paired *t*-test. Data are mean±SEM.

Fig. S2

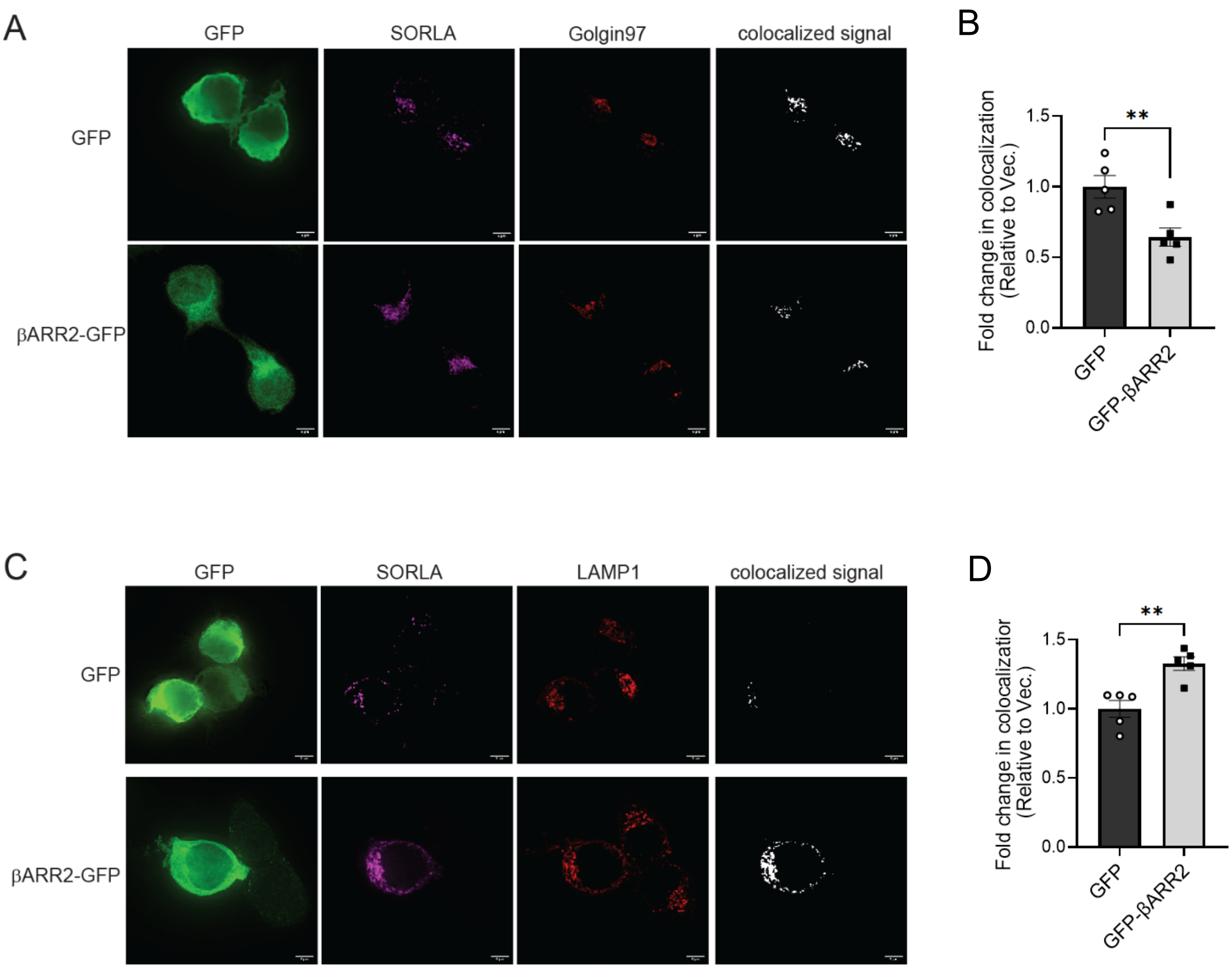

Fig. S2. Overexpression of βARR2 reduces SORLA localization in TGN while increasing it in late endosomes/lysosomes. Neuro2A cells co-expressing Myc-SORLA with βARR2-GFP or GFP were subjected to immunostaining. Representative images (A,C) and quantification of colocalization coefficient (B,D) of SORLA with Golgin97 or LAMP1. Scale bar, 5μm. **, p<0.01; ***, p<0.001 by unpaired t-test. Data are mean±SEM.

Fig. S3

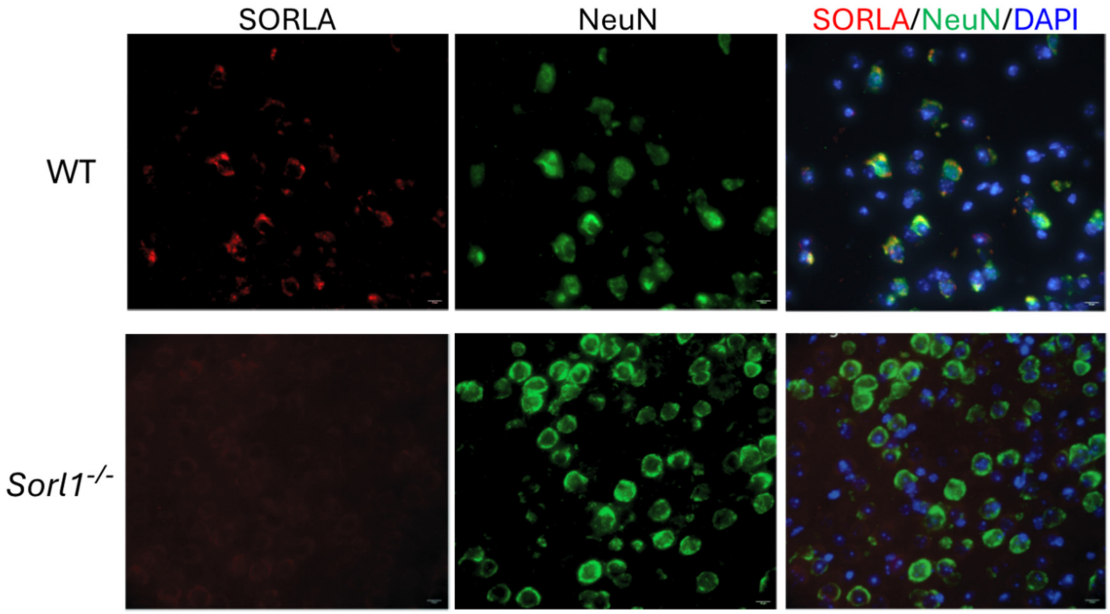

Fig. S3. Validation of SORLA antibody for immunostaining. Cortical sections prepared from WT or *Sorl1*$^{-/-}$ mice were subjected to immunostaining using a rabbit anti-SORLA antibody (Protein Tech) and a mouse anti-NeuN antibody (Sigma). Scale bar, 5µm.

Fig. S4

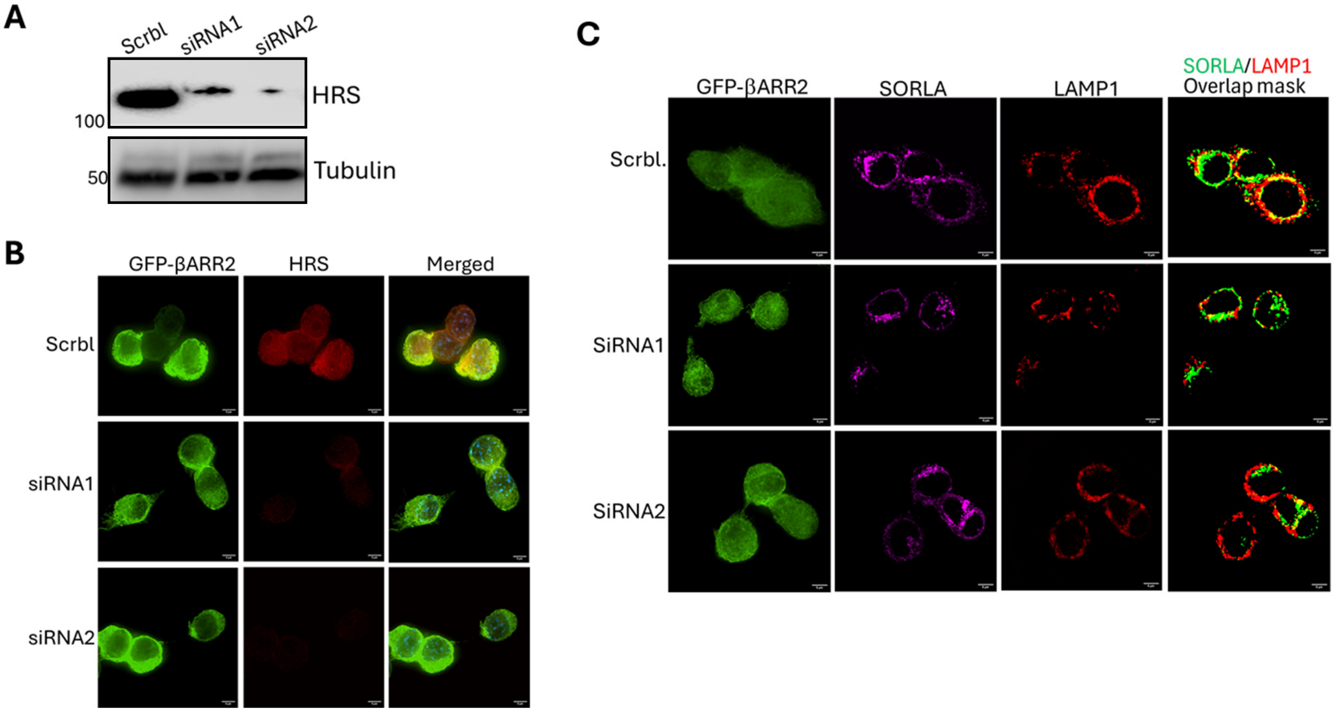

Fig. S4. Knocking down *Hrs* reduces SORLA localization in late endosomes/lysosomes. Neuro2A cells stably expressing SNAP-SORLA were co-transfected with GFP-βARR2 and siRNA against Hrs or scrambled siRNA. Cells were subjected to immunostaining 48 hrs post-transfection.. Representative Western blot (A) and immunostaining images (B) showing successful knockdown of HRS expression. (C) Representative immunostaining images showing SORLA colocalization with the late endosomal/lysosomal marker, LAMP1. Scale bar, 5 µm. Quantification is shown in Fig. 3F.

Fig. S5

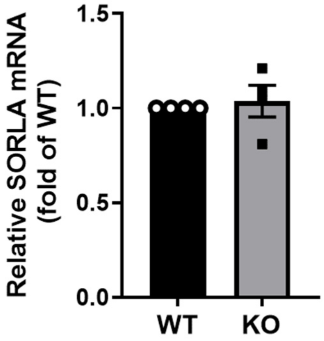

Fig. S5. The mRNA level of SORLA is not changed in βARR2 deficient mice. Cortices from WT or *Arr2b*$^{-/-}$ (KO) mice were subjected to RNA extraction and qRT-PCR. No significant difference was detected using a paired *t*-test. Data are mean±SEM.

Fig. S6

A

Empty Vector:
LQSSFTAFANSHYSSR, Charge: +2, q value =1.6e-4

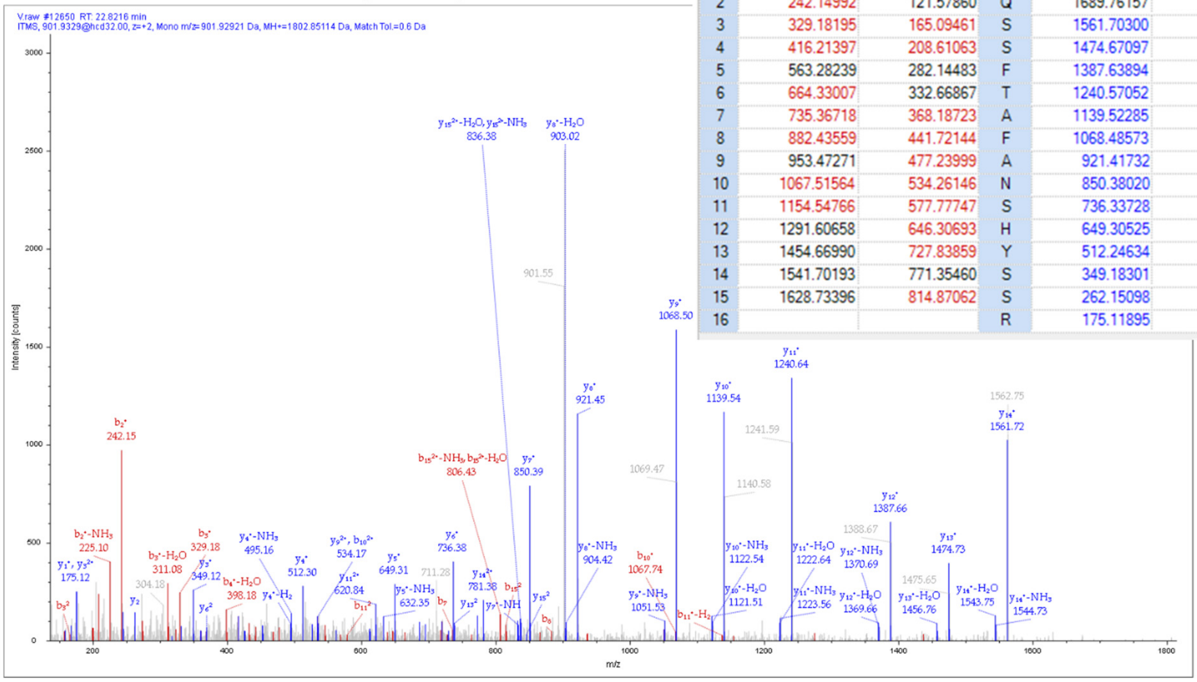

#1	b^+	b^{2+}	Seq.	y^+	y^{2+}	#2
1	114.09134	57.54931	L			16
2	242.14992	121.57860	Q	1689.76157	845.38442	15
3	329.18195	165.09461	S	1561.70300	781.35514	14
4	416.21397	208.61063	S	1474.67097	737.83912	13
5	563.28239	282.14483	F	1387.63894	694.32311	12
6	664.33007	332.66867	T	1240.57052	620.78890	11
7	735.36718	368.18723	A	1139.52285	570.26506	10
8	882.43559	441.72144	F	1068.48573	534.74650	9
9	953.47271	477.23999	A	921.41732	461.21230	8
10	1067.51564	534.26146	N	850.38020	425.69374	7
11	1154.54766	577.77747	S	736.33728	368.67228	6
12	1291.60658	646.30693	H	649.30525	325.15626	5
13	1454.66990	727.83859	Y	512.24634	256.62681	4
14	1541.70193	771.35460	S	349.18301	175.09514	3
15	1628.73396	814.87062	S	262.15098	131.57913	2
16			R	175.11895	88.06311	1

B

PKCι/λ^{Cat}:
LQSSFTAFANSHYSsR, S15-Phospho (79.96633 Da)
Charge: +2, q value=5.5e-4

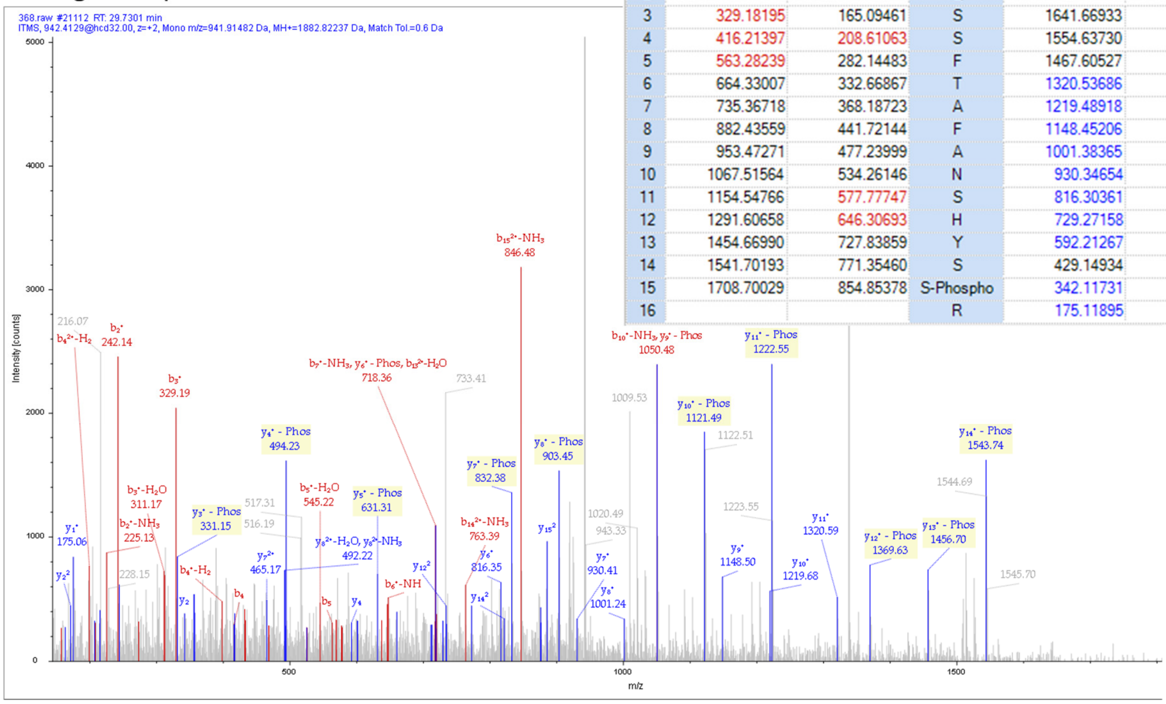

#1	b^+	b^{2+}	Seq.	y^+	y^{2+}	#2
1	114.09134	57.54931	L			16
2	242.14992	121.57860	Q	1769.72790	885.36759	15
3	329.18195	165.09461	S	1641.66933	821.33830	14
4	416.21397	208.61063	S	1554.63730	777.82229	13
5	563.28239	282.14483	F	1467.60527	734.30627	12
6	664.33007	332.66867	T	1320.53686	660.77207	11
7	735.36718	368.18723	A	1219.48918	610.24823	10
8	882.43559	441.72144	F	1148.45206	574.72967	9
9	953.47271	477.23999	A	1001.38365	501.19546	8
10	1067.51564	534.26146	N	930.34654	465.67691	7
11	1154.54766	577.77747	S	816.30361	408.65544	6
12	1291.60658	646.30693	H	729.27158	365.13943	5
13	1454.66990	727.83859	Y	592.21267	296.60997	4
14	1541.70193	771.35460	S	429.14934	215.07831	3
15	1708.70029	854.85378	S-Phospho	342.11731	171.56229	2
16			R	175.11895	88.06311	1

C

PKCι/λ^A120E:
LQSSFTAFANSHYSsR, S15-Phospho (79.96633 Da)
Charge: +2, q value=3.6e-3

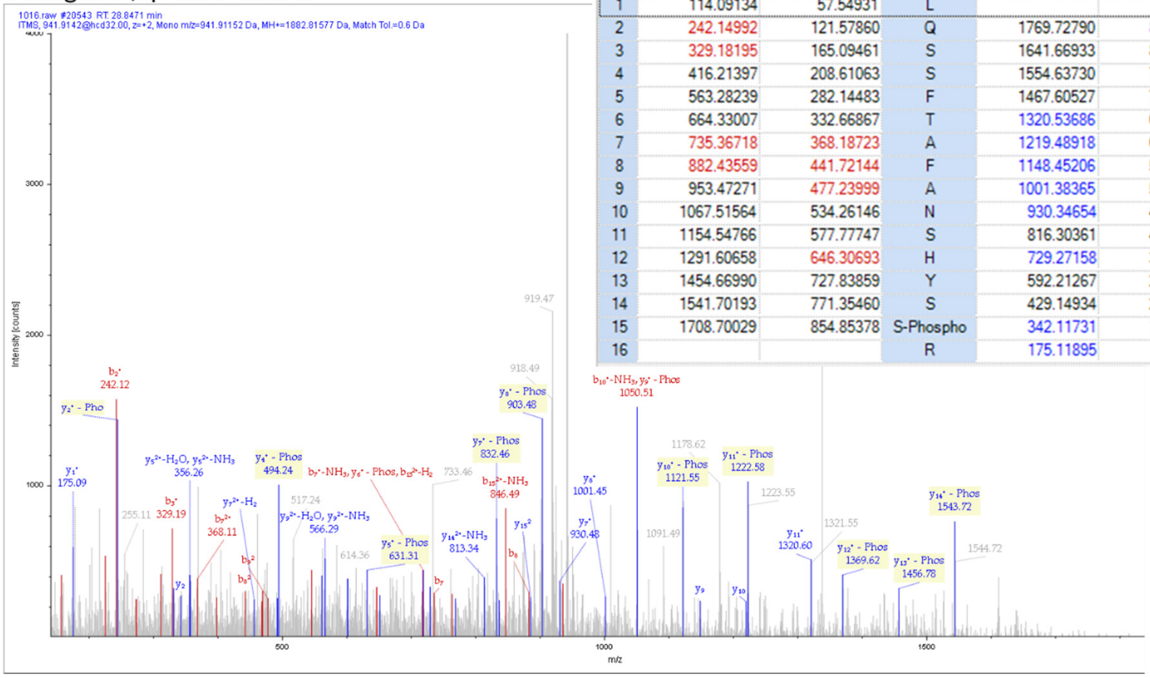

#1	b+	b2+	Seq.	y+	y2+	#2
1	114.09134	57.54931	L			16
2	242.14992	121.57860	Q	1769.72790	885.36759	15
3	329.18195	165.09461	S	1641.66933	821.33830	14
4	416.21397	208.61063	S	1554.63730	777.82229	13
5	563.28239	282.14483	F	1467.60527	734.30627	12
6	664.33007	332.66867	T	1320.53686	660.77207	11
7	735.36718	368.18723	A	1219.48918	610.24823	10
8	882.43559	441.72144	F	1148.45206	574.72967	9
9	953.47271	477.23999	A	1001.38365	501.19546	8
10	1067.51564	534.26146	N	930.34654	465.67691	7
11	1154.54766	577.77747	S	816.30361	408.65544	6
12	1291.60658	646.30693	H	729.27158	365.13943	5
13	1454.66990	727.83859	Y	592.21267	296.60997	4
14	1541.70193	771.35460	S	429.14934	215.07831	3
15	1708.70029	854.85378	S-Phospho	342.11731	171.56229	2
16			R	175.11895	88.06311	1

D

PKCι/λ^A120E:
LQSSFTAFANSHYsSR, S14-Phospho (79.96633 Da)
Charge: +2, q value=1.5e-4

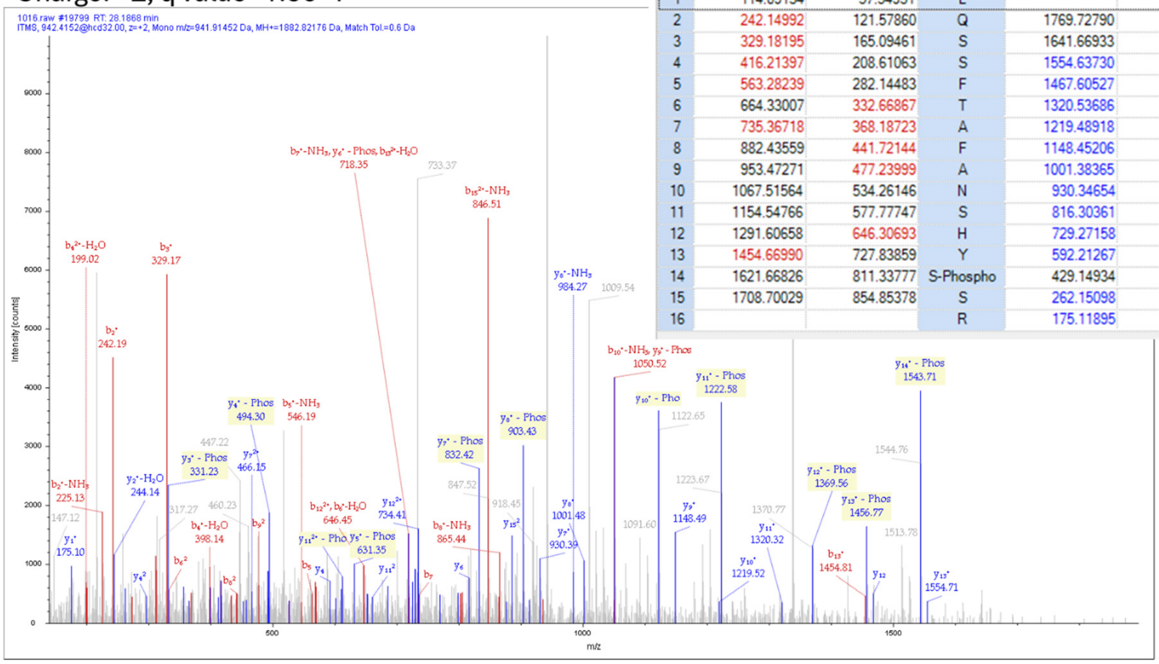

#1	b+	b2+	Seq.	y+	y2+	#2
1	114.09134	57.54931	L			16
2	242.14992	121.57860	Q	1769.72790	885.36759	15
3	329.18195	165.09461	S	1641.66933	821.33830	14
4	416.21397	208.61063	S	1554.63730	777.82229	13
5	563.28239	282.14483	F	1467.60527	734.30627	12
6	664.33007	332.66867	T	1320.53686	660.77207	11
7	735.36718	368.18723	A	1219.48918	610.24823	10
8	882.43559	441.72144	F	1148.45206	574.72967	9
9	953.47271	477.23999	A	1001.38365	501.19546	8
10	1067.51564	534.26146	N	930.34654	465.67691	7
11	1154.54766	577.77747	S	816.30361	408.65544	6
12	1291.60658	646.30693	H	729.27158	365.13943	5
13	1454.66990	727.83859	Y	592.21267	296.60997	4
14	1621.66826	811.33777	S-Phospho	429.14934	215.07831	3
15	1708.70029	854.85378	S	262.15098	131.57913	2
16			R	175.11895	88.06311	1

Fig. S6. PKCι/λ activation induces SORLA phosphorylation at ^{2178}SerSer^{2179}. HEK cells were co-transfected with a plasmid encoding Myc-SORLA and a 2nd plasmid encoding HA-tagged PKCι/λ^{Cat} or V5-tagged PKCι/λ^{A120E}. An empty vector was used as the second plasmid for the negative control. Prior to harvest, cells were treated with Calyculin A (50 nM for 15 minutes) to enhance phosphorylation signals. Lysates were subjected to IP using anti-Myc magnetic beads and IP'ed samples were analyzed by mass spectrometry for phosphorylation site identification. Spectra containing the peptide of interest obtained from cells transfected with different constructs are shown. "-Phos" refers to loss of a phosphate group and water (-H_3PO_4).

Fig. S7

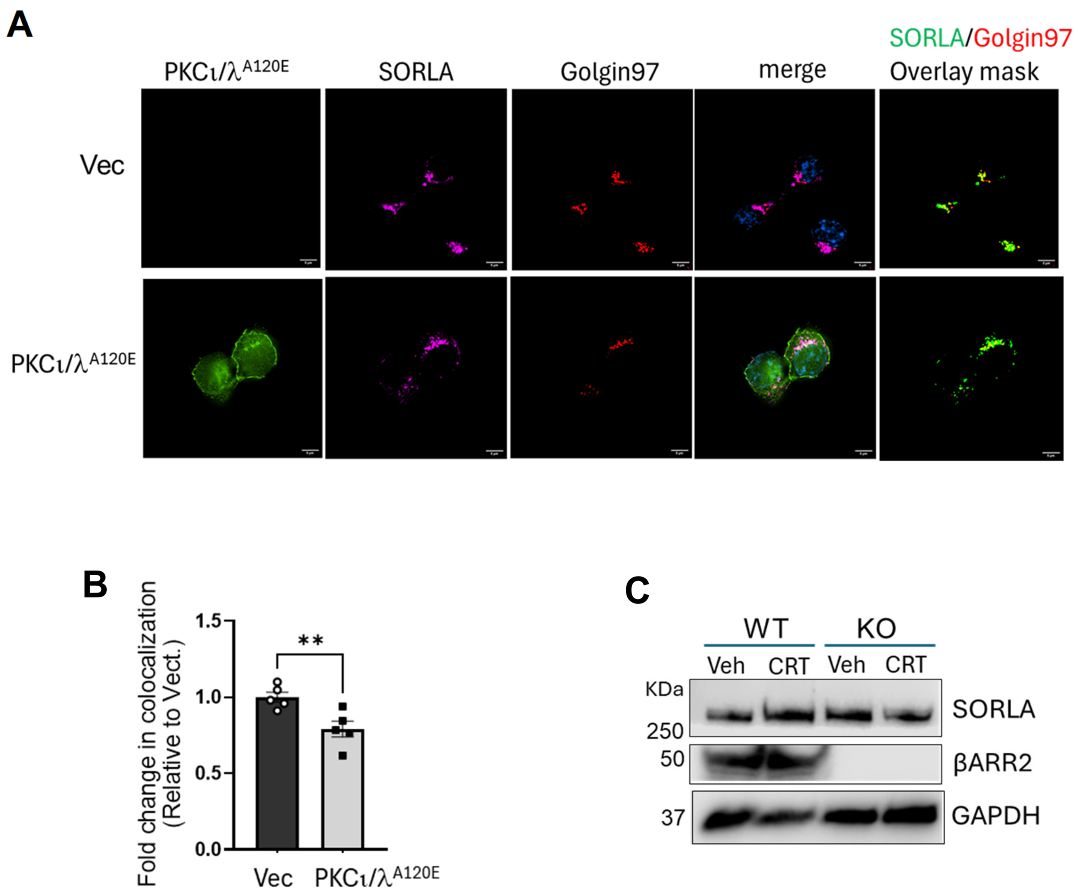

Fig. S7. Overexpression of constitutively active PKCι/λ reduces SORLA localization in the TGN. (A&B) Neuro2A cells co-expressing Myc-SORLA and V5-PKCι/λ^{A120E} were subjected to immunostaining. Representative images (A) and quantification of SORLA colocalization with Golgin97 (B) are shown. **, p<0.01by unpaired t test. Scale bar, 5 µm. **(C)** Representative blots of SORLA and βARR2 in cortical lysates prepared from WT and *Arr2b*$^{-/-}$ brains with indicated treatment. Quantifications are shown in Fig. 5H and I.

Fig. S8

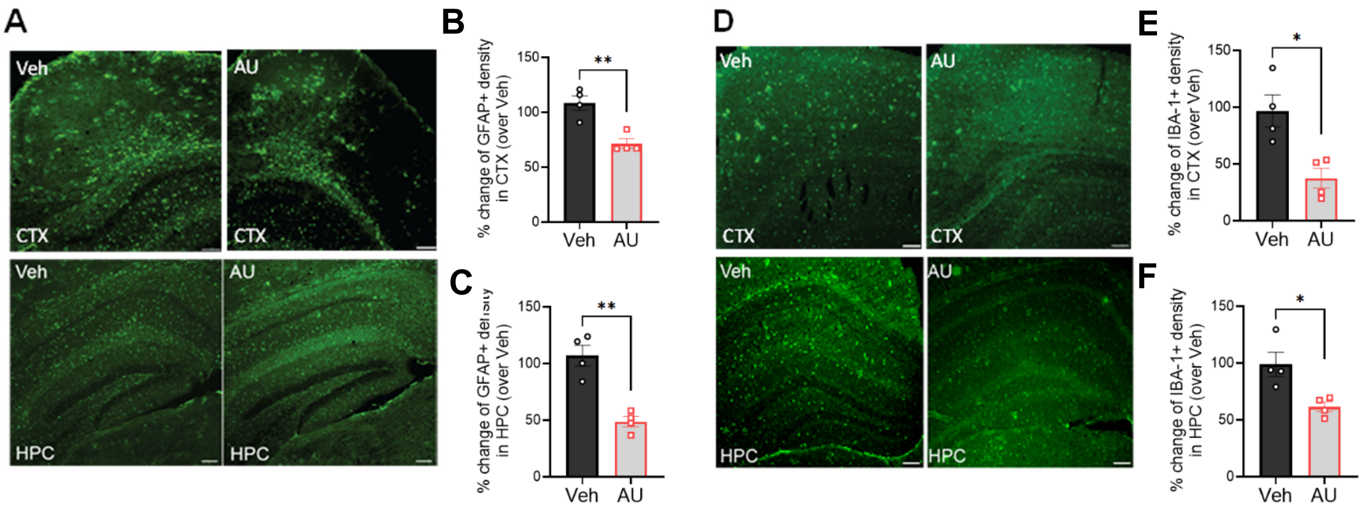

Fig. S8. Auranofin treatment reduces astrocyte and microglia reactivity in AppKI mice. Brain sections from vehicle (Veh) or auranofin (AU) treated mice were subjected to immunostaining using a GFAP or IBA-1 antibody. **(A and D)** Representative images of GFAP (A) and IBA-1 (D) in the cortex (CTX) and hippocampus (HPC). **(B&C)** Quantification of the density of GFAP+ cells in the cortex (B) and hippocampus (C). **(E&F)** Quantification of the density change in IBA-1+ cells in the cortex (E) and hippocampus (F). Scale bar, 100 µm. *, $p<0.05$; **, $p<0.01$; ***, $p<0.001$ by unpaired *t*-test. Data are mean±SEM.

Fig. S9

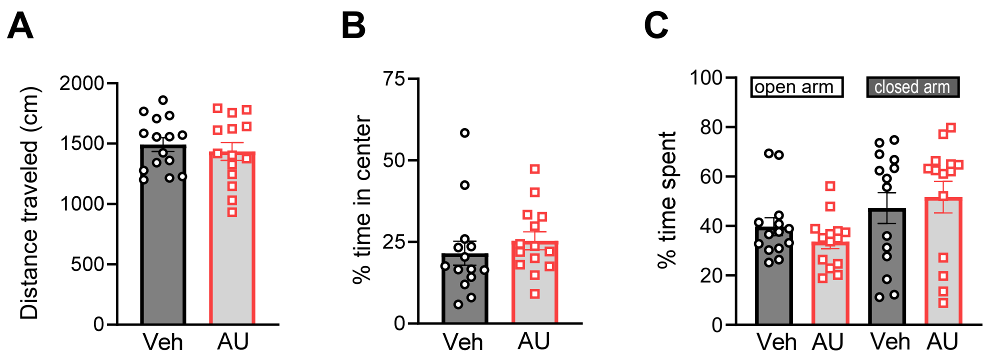

Fig. S9. Auranofin treatment shows no effect on general activity or anxiety-like behaviors in AppKI mice. Mice were evaluated in the open field (A&B) and elevated zero maze (C) tests. **(A)** Quantification of total distance traveled in the open field test. **(B)** Quantification of the time spent in the center of the open field. **(C)** Quantification of the time spent in open or closed arms. Data are mean±SEM.

Fig. S10

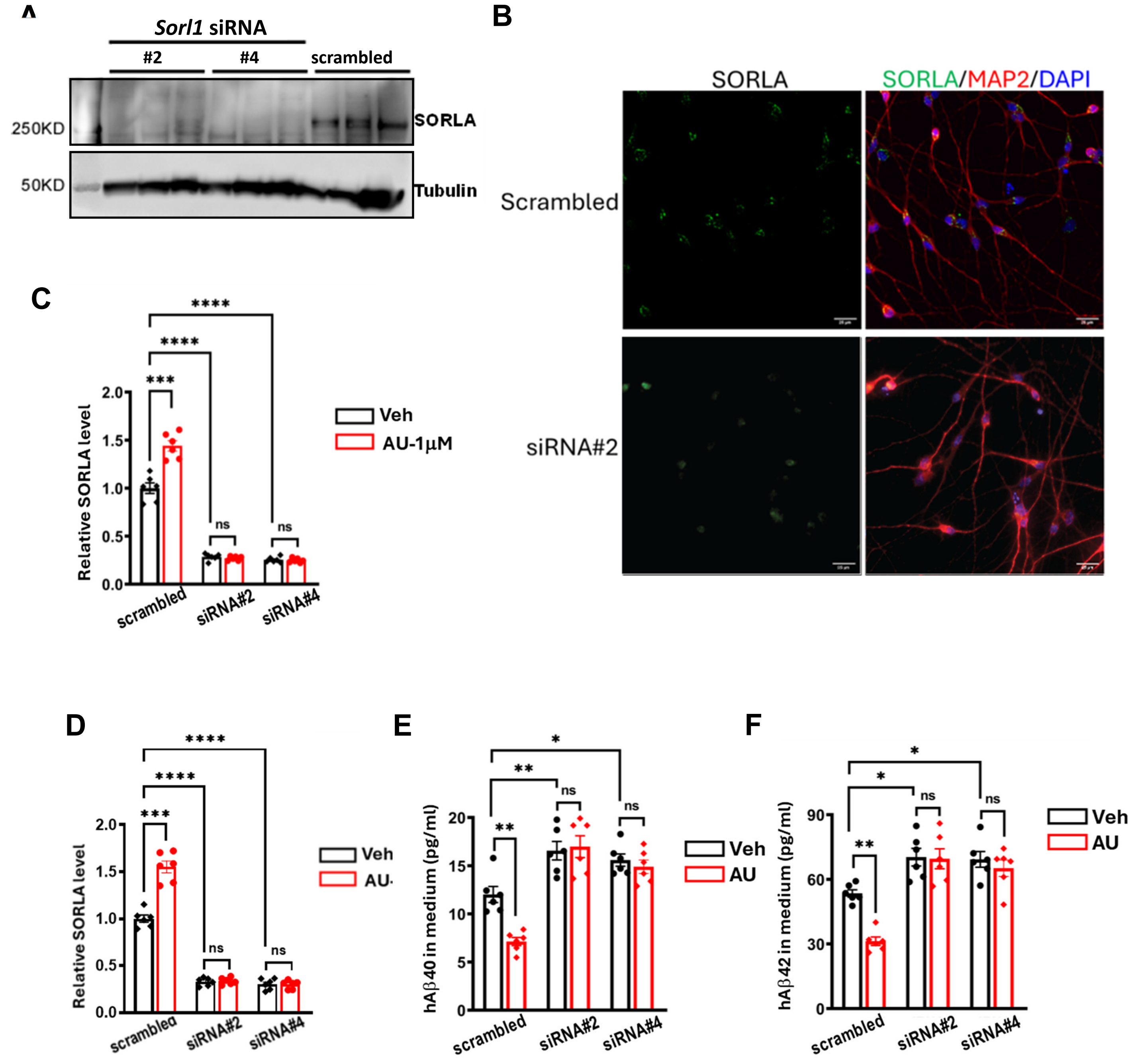
A
Sorl1 siRNA
#2
#4
scrambled
250KD
SORLA
50KD
Tubulin
B
SORLA
SORLA/MAP2/DAPI
Scrambled
siRNA#2
C
Relative SORLA level
Veh
AU-1μM
scrambled
siRNA#2
siRNA#4
D
Relative SORLA level
Veh
AU
E
hAβ40 in medium (pg/ml)
F
hAβ42 in medium (pg/ml)

Fig. S10. The effect of auranofin on Aβ generation relies on SORLA expression. hiPSCs-derived cortical neurons were transfected with different siRNAs against *SORL1* or scrambled siRNA and treated with auranofin (AU, 1 μM) or vehicle (Veh) for 72 hrs. **(A)** Representative Western blots of total lysates with indicated siRNA transfection from neurons derived from control iPSCs (line EX-SeV-CW50065). **(B)** Representative immunostaining images of siRNA knockdown in neurons derived from AD iPSCs (line EX-SeV-CW50114). Scale bar, 25 μm. **(C&D)** Quantification of SORLA in neurons derived from AD iPSCs (line EX-SeV-CW50114, C) and AD iPSC line (EX-SeV-CW50137, D), respectively. **(E&F)** Quantification of human Aβ40 (E) and Aβ42 (F) in culture medium measured by ELISA. *, $p<0.05$; **, $p<0.01$; ***, $p<0.001$; ****, $p<0.0001$ by two-way ANOVA Sadak's multiple comparisons test. Data are mean±SEM.

Table S1. Commercial antibodies used in this study.

S.No	Target	Species	Company	Catalogue Number	Application	Figure Panel
1	GST	Goat	Sigma	GE27-4577-01	Western blot	1A
2	GFP	Chicken	Abcam	13970	Western blot	1C, 1D, 1E, 3A, 4A, 4C, S4B
3	Myc	Mouse	Cell Signaling	2276S	Co-IP, Western blot	1C, 1D, 1E, 4A, 4C, S1A
4	Myc	Rabbit	Cell Signaling	2278S	Co-IP, Western blot	3A, 4G, 4J
5	FLAG	Rabbit	Sigma	F7425	Western blot	1D, 1E, S1A
6	SORLA	Rabbit	Proteintech	22592-1-AP	Co-IP, Western blot, IHC	2A, 2C, 2E, 3C, 3E, 3G, 3I, 3K, 4E, 5A, 5C, 5E, 5G, 6A, 7C, S3, S4B, S4C, S9A, S9B
7	SORLA	Mouse	Sigma	MABN1793	Co-IP, Western blot	1G, 6D
8	VPS35	Goat	Origene	TA302699	Western blot	1G, 1I, S1A
9	β-Arrestin 2	Rabbit	Fisher	PA1-732	Western blot	3K, 6D
10	β-Arrestin 2	Goat	Abcam	ab31294	Western blot	3C, 4E
11	Golgin 97	Mouse	Fisher	A21270	IHC	2A
12	Lamp1	Rat	DSHB	1D4B	IHC	2C, 3E, 5A, S4C
13	EEA1	Mouse	BD Biosciences	610457	IHC	2E
14	Hrs	Mouse	Santa Cruz	271455	Western blot	3A, 3C, S4A, S4B
15	GAPDH	HRP	Proteintech	HRP-60004	Western blot	3G, 3K, 5E, 5G, 6F, 7A
16	V5-Tag (D3H8Q)	Rabbit	Cell signaling	13202	Western blot	4C
17	p-Serine	Rabbit	Abcam	ab9332	Western blot	4G, 4J
18	HA.11	Mouse	Biolegend Inc.	901515	Western blot	4G, 4J
19	HA	Rat	Roche	11867431001	IHC	5A
20	Tubulin	Rabbit	Proteintech	10094-1-AP	Western blot	3I, 5C, S4A, S9A
21	PKCi/l	Rabbit	Fisher	MA5-14874	Western blot	5G, 6A, 7A
22	pPKCi/l	Rabbit	Fisher	PI700582	Western blot	5G, 6A, 7A
23	Actin	HRP-conjugated	Fisher	HRP-60008	Western blot	6A
24	APP-FL	Rabbit	Abcam	ab32136	Western blot	6F
25	sAPPβ	Rabbit	IBL	18957	Western blot	6F
26	β-CTF (6E10)	Mouse	Biolegend	Previously Covance catalog# SIG-39320	Western blot	6F
27	Aβ	Mouse	Abcam	ab11132	IHC	6K
28	MAP2	Chicken	Fisher	PA1-10005	IHC	7C, S9B
29	NeuN	Mouse	Sigma	MAB377	IHC	S3
30	GFAP	Rabbit	Abclonal	A14673	IHC	S7A
31	IBA-1	Rabbit	Abcam	ab178847	IHC	S7D
32						

Table S2. Information of human samples used in this study.

	autopsy Number	age	sex	AP_PMI	calc_ braakStage	CERAD
Ctrl.	11-101	87	Male	6	0 (B0)	No neuritic plaques (C0)
	12-073	96	Female	6.32	I (B1)	Sparse (C1)
	15-051	70	Female	4.17	I (B1)	Sparse (C1)
	15-056	74	Female	4.5	I (B1)	Sparse (C1)
	11-070	101	Male	4.33	II (B1)	No neuritic plaques (C0)
	12-072	83	Male	7	II (B1)	No neuritic plaques (C0)
	10-071	95	Male	7.5	II (B1)	No neuritic plaques (C0)
	13-039	84	Male	4.5	II (B1)	No neuritic plaques (C0)
	14-052	73	Male	4.5	II (B1)	No neuritic plaques (C0)
	13-002	86	Male	3.25	III (B2)	No neuritic plaques (C0)
	06-101	87	Male	7.5	III (B2)	No neuritic plaques (C0)
AD	13-054	84	Female	4.47	V (B3)	Frequent (C3)
	10-092	76	Male	3.92	V (B3)	Frequent (C3)
	07-014	67	Female	4.75	VI (B3)	Frequent (C3)
	07-076	87	Female	5.27	VI (B3)	Frequent (C3)
	07-085	59	Male	5.08	VI (B3)	Frequent (C3)
	08-073	91	Female	5.75	VI (B3)	Frequent (C3)
	09-117	88	Male	4.38	VI (B3)	Frequent (C3)
	12-082	62	Male	3.92	VI (B3)	Frequent (C3)
	14-084	77	Male	7.25	VI (B3)	Frequent (C3)
	11-025	88	Male	4.67	VI (B3)	Moderate (C2)
	08-046	83	Male	8.67	VI (B3)	Frequent (C3)
