# Supplementary material for: The PKCι-β-arrestin2 axis disrupts SORLA retrograde trafficking, driving its degradation and amyloid pathology in Alzheimer’s disease
Source: Mol Neurodegener. 2025 Jun 23;20:76. doi: 10.1186/s13024-025-00865-6 (PMC12186327; doi:10.1186/s13024-025-00865-6)
Supplement: Supplementary file 2 — Supplementary Material 2. [file 13024_2025_865_MOESM2_ESM.pdf]

S.No	Target	Species	Company	Catalogue Number	Application	Figure Panel
1	GST	Goat	Sigma	GE27-4577-01	Western blot	1A
2	GFP	Chicken	Abcam	13970	Western blot	1C, 1D, 1E, 3A, 4A, 4C, S4B
3	Myc	Mouse	Cell Signaling	2276S	Co-IP, Western blot	1C, 1D, 1E, 4A, 4C, S1A
4	Myc	Rabbit	Cell Signaling	2278S	Co-IP, Western blot	3A, 4G, 4J
5	FLAG	Rabbit	Sigma	F7425	Western blot	1D, 1E, S1A
6	SORLA	Rabbit	Proteintech	22592-1-AP	Co-IP, Western blot, IHC	2A, 2C, 2E, 3C, 3E, 3G, 3I, 3K, 4E, 5A, 5C, 5E, 5G, 6A, 7C, S3, S4B, S4C, S9A, S9B
7	SORLA	Mouse	Sigma	MABN1793	Co-IP, Western blot	1G, 6D
8	VPS35	Goat	Origene	TA302699	Western blot	1G, 1I, S1A
9	β-Arrestin 2	Rabbit	Fisher	PA1-732	Western blot	3K, 6D
10	β-Arrestin 2	Goat	Abcam	ab31294	Western blot	3C, 4E
11	Golgin 97	Mouse	Fisher	A21270	IHC	2A
12	Lamp1	Rat	DSHB	1D4B	IHC	2C, 3E, 5A, S4C
13	EEA1	Mouse	BD Biosciences	610457	IHC	2E
14	Hrs	Mouse	Santa Cruz	271455	Western blot	3A, 3C, S4A, S4B
15	GAPDH	HRP	Proteintech	HRP-60004	Western blot	3G, 3K, 5E, 5G, 6F, 7A
16	V5-Tag (D3H8Q)	Rabbit	Cell signaling	13202	Western blot	4C
17	p-Serine	Rabbit	Abcam	ab9332	Western blot	4G, 4J, 4K
18	HA.11	Mouse	Biolegend Inc.	901515	Western blot	4G, 4J
19	HA	Rat	Roche	11867431001	IHC	5A
20	Tubulin	Rabbit	Proteintech	10094-1-AP	Western blot	3I, 5C, S4A, S9A
21	PKCi/l	Rabbit	Fisher	MA5-14874	Western blot	5G, 6A, 7A
22	pPKCi/l	Rabbit	Fisher	PI700582	Western blot	5G, 6A, 7A
23	Actin	HRP-conjugated	Fisher	HRP-60008	Western blot	6A
24	APP-FL	Rabbit	Abcam	ab32136	Western blot	6F
25	sAPPβ	Rabbit	IBL	18957	Western blot	6F
26	β-CTF (6E10)	Mouse	Biolegend	Previously Covance catalog# SIG-39320	Western blot	6F
27	Aβ	Mouse	Abcam	ab11132	IHC	6K
28	MAP2	Chicken	Fisher	PA1-10005	IHC	7C, S9B
29	NeuN	Mouse	Sigma	MAB377	IHC	S3
30	GFAP	Rabbit	Abclonal	A14673	IHC	S7A
31	IBA-1	Rabbit	Abcam	ab178847	IHC	S7D
32	pSer	Rabbit	Cell Signaling	Antibody #9631-	Western blot	4K
